# Probiotic supplementation during antibiotic treatment is unjustified in maintaining the gut microbiome diversity: a systematic review and meta-analysis

**DOI:** 10.1186/s12916-023-02961-0

**Published:** 2023-07-19

**Authors:** Anna Júlia Éliás, Viktória Barna, Cristina Patoni, Dóra Demeter, Dániel Sándor Veres, Stefania Bunduc, Bálint Erőss, Péter Hegyi, László Földvári-Nagy, Katalin Lenti

**Affiliations:** 1 Centre for Translational Medicine, Semmelweis University, Budapest, Hungary; 2 Doctoral School of Health Sciences, Semmelweis University, Budapest, Hungary; 3 Faculty of Health Sciences, Semmelweis University, Budapest, Hungary; 4 Carol Davila University of Medicine and Pharmacy, Bucharest, Romania; 5 Military Hospital Medical Centre, Hungarian Defense Forces, Budapest, Hungary; 6 Department of Biophysics and Radiation Biology, Semmelweis University, Budapest, Hungary; 7 Fundeni Clinical Institute, Bucharest, Romania; 8 Institute of Pancreatic Diseases, Semmelweis University, Budapest, Hungary; 9 Institute for Translational Medicine, Medical School, University of Pécs, Pécs, Hungary; 10 Department of Morphology and Physiology, Faculty of Health Sciences, Semmelweis University, Budapest, Hungary

**Keywords:** Antibiotics, Probiotics, Gastrointestinal microbiome, Meta-analysis, Diversity

## Abstract

**Background:**

Probiotics are often used to prevent antibiotic-induced low-diversity dysbiosis, however their effect is not yet sufficiently summarized in this regard. We aimed to investigate the effects of concurrent probiotic supplementation on gut microbiome composition during antibiotic therapy.

**Methods:**

We performed a systematic review and meta-analysis of randomized controlled trials reporting the differences in gut microbiome diversity between patients on antibiotic therapy with and without concomitant probiotic supplementation. The systematic search was performed in three databases (MEDLINE (via PubMed), Embase, and Cochrane Central Register of Controlled Trials (CENTRAL)) without filters on 15 October 2021. A random-effects model was used to estimate pooled mean differences (MD) with 95% confidence intervals (CI). This review was registered on PROSPERO (CRD42021282983).

**Results:**

Of 11,769 identified articles, 15 were eligible in the systematic review and 5 in the meta-analyses. Quantitative data synthesis for Shannon (MD = 0.23, 95% CI: [(−)0.06–0.51]), Chao1 (MD = 11.59 [(−)18.42–41.60]) and observed OTUs (operational taxonomic unit) (MD = 17.15 [(−)9.43–43.73]) diversity indices revealed no significant difference between probiotic supplemented and control groups. Lacking data prevented meta-analyzing other diversity indices; however, most of the included studies reported no difference in the other reported α- and ß-diversity indices between the groups. Changes in the taxonomic composition varied across the eligible studies but tended to be similar in both groups. However, they showed a potential tendency to restore baseline levels in both groups after 3–8 weeks.

This is the first meta-analysis and the most comprehensive review of the topic to date using high quality methods. The limited number of studies and low sample sizes are the main limitations of our study. Moreover, there was high variability across the studies regarding the indication of antibiotic therapy and the type, dose, and duration of antimicrobials and probiotics.

**Conclusions:**

Our results showed that probiotic supplementation during antibiotic therapy was not found to be influential on gut microbiome diversity indices. Defining appropriate microbiome diversity indices, their standard ranges, and their clinical relevance would be crucial.

**Supplementary Information:**

The online version contains supplementary material available at 10.1186/s12916-023-02961-0.

## Background

Antibiotic treatment affects the gut bacterial microbiota quantitatively and qualitatively, causing a decrease or even extinction of certain species, leading to a low-diversity microbiome, and allowing some potentially harmful bacteria to become dominant, e.g., *Clostridium perfringens*, *Staphylococcus aureus*, or *Clostridioides difficile* [1, 2]. This microbial imbalance is called dysbiosis. The deviation from the normal microbiome has been linked to obesity, malnutrition, inflammatory bowel disease, neurological dysfunctions, and cancer [3]. The gut microbiota can spontaneously recover, but it is influenced by various host and external factors like age, health status, the geographical area of origin of patients, dose, duration, and the spectrum of antibiotic treatment [4–6]. Young, healthy adults have stable microbial community functions [7], but repeated perturbation of the ecosystem is particularly detrimental if there is insufficient time for recovery after the initial impairment. Previous research has shown that the gut microbiota recovers within about 2 weeks after a single antibiotic exposure in adults, but repeated exposures can significantly prolong the recovery time [4, 8–10].

Probiotics are preparations containing live micro-organisms, typically composed of microbes that are also found in the natural gut flora. Probiotics may contain bacteria, yeasts, or a mixture of them [11]. These products are used to prevent dysbiosis; however, the effects of concurrent probiotic supplementation on fecal microbiota diversity and taxonomical composition during antibiotic therapy are not fully understood. The effects of these products on clinical outcomes during antibiotic therapy have been intensely researched; however, most research did not focus on investigating the composition of the gut microbiome. This aspect is also missing from the current guidelines on the use of probiotics of the American Gastroenterological Association (AGA) and World Gastroenterology Organization (WGO) [11, 12].

So far, reported results have been highly variable both in terms of the reported outcomes and the conclusion about the efficacy of probiotics on microbiota restoration after antibiotic therapy. The biggest challenges in analyzing it are the lack of a consensus definition for ‘normal’ microbiota, generally accepted diversity indicators, and standard measurement methods [13–16]. Moreover, the significant inter-individual variation in microbial species makes it difficult to define the normal microbiota. Several types of diversity indices have been described so far [17]. Diversity indices commonly used in ecology are used to characterize microbiome diversity. α-diversity indices reflect the diversity of a single sample, measuring species richness (number of species) and/or distribution (evenness of species). Each alpha diversity index is calculated differently, depending on factors like how the presence or absence of certain rare species is assessed and interpreted. In contrast, β-diversity indices can be used to compare different samples and communities. It can consider both the overall abundance per sample and the abundance of each taxon [17]. In simple terms, α-diversity represents a within-sample diversity, whereas β-diversity describes similarity or dissimilarity between samples [3, 14, 15].

We aimed to systematically review and meta-analyze the effect of probiotics on antibiotic-induced dysbiosis in randomized controlled trials.

## Methods

We designed the study according to the Cochrane recommendations [18]. We previously submitted our study protocol to the International prospective register of systematic reviews (PROSPERO) (CRD42021282983**)** and applied it consistently (Additional File 1: Supplementary Methods S1). When reporting our results, we followed the guidance of the Preferred Reporting Items for Systematic Reviews and Meta-Analyses (PRISMA) 2020 Statement [19] (Additional File 2: Table S1).

### Systematic search and selection

The PICO-S format (population, intervention, comparison, outcome, and study design) was used to formulate our clinical question and establish the eligibility criteria. We included all the studies that met the following eligibility criteria: population (P) — people treated with antibiotics regardless of indication; intervention (I) — probiotic supplementation along with antibiotic treatment; comparison group (C) — no probiotic supplementation. The assessed outcomes (O) were gut microbial diversity and composition (any diversity indices reported) at the end of the intervention and after a follow-up period, as reported in each study. No restrictions were applied regarding sex, age, ethnicity, or associated comorbidities. Only randomized controlled trials (RCTs) were included.

The systematic search was conducted without filters or restrictions in three medical databases — MEDLINE (via PubMed), Embase, and Cochrane Central Register of Controlled Trials (CENTRAL) using the search key detailed in Additional File 1: Supplementary Methods S2 until 15 October 2021. We manually screened the reference lists of the studies included in the review for additional eligible articles. If our search did not retrieve the published protocols for the identified eligible studies, we tried to find them at https://www.clinicaltrialsregister.eu/ and https://clinicaltrials.gov/.

The selection was performed with the reference management program EndNote X9 (Clarivate Analytics, Philadelphia, PA, USA). After automatic and manual duplicate removal, two independent investigators manually selected the articles stepwise, first by title and abstract and subsequently by full-text contents adhering to the predefined eligibility criteria. Cohen’s kappa coefficient was calculated at each selection step to quantify the agreement between assessors. Disagreements were solved by consensus.

### Data collection

Two independent authors (AJÉ and VB) extracted the data in each article manually and crosschecked each other’s data pool. Disagreements were solved by consensus. The information was summarized in a standardized data collection form (Microsoft Excel, Microsoft Office 365, Redmond, WA, USA). The following data were extracted: study characteristics (first author, year of publication, country, number of centers, and setting), population description (sample size, sex distribution, age, and indication for antibiotic therapy), therapy details for both probiotics and antibiotics (drug/probiotic type, dose, and duration), and outcomes as reported in each article. Outcomes are detailed in Additional File 2: Table S2 [20–36]. When data were available in graphic format only, we performed the extraction with GetData Graph Digitizer software (v. 2.26.0.20.) [37].

### Synthesis methods

The statistical analysis was performed by a biostatistician using the *R* software [38] with *meta* [39] and *dmetar* [40] packages. A meta-analysis was performed if the evaluated outcome was reported in at least three articles. For the effect size measure, we calculated mean differences (MD, probiotic and antibiotic minus only antibiotic treatment) with 95% confidence intervals (CIs). The mean and the corresponding standard deviations (SD) were extracted from each study if available. In other cases, to estimate the mean and standard deviation based on 0,1,2,3,4 quartiles (extracted from box plots), Luo [41] and Shi [42] methods were used as implemented in the *meta* package. On the basis of the article of Oh et al. [43], where the raw data of Shannon, Chao1, and observed OTUs (operational taxonomic units) diversity indices were given, we could assume that the distribution of these indices did not differ from a normal distribution in relevant amount, and therefore the estimation of mean and SD from the quantiles could be acceptable. As the main result, we pooled the values of Shannon, Chao1, and observed OTUs diversity indices after treatment and used the inverse variance weighting method to each of them separately. We included only RCTs; therefore, we could assume that the characteristics before the treatment were not different in the intervention and control groups. As an additional sensitivity analysis, we performed a separate analysis for data before the treatment and a meta-analysis for the “before-after” change values. For the change calculations, we used the correlation coefficient determined from the data of Oh et al. [43]. As we anticipated considerable between-study heterogeneity, a random-effects model was used to pool the effect sizes. We did not apply the Hartung-Knapp adjustment [44, 45]. The maximum-likelihood estimator was applied with the Q profile method for confidence interval to estimate the heterogeneity variance measure *τ* ^2^ [46]. Additionally, between-study heterogeneity was described using the Cochran’s *Q* test and Higgins&Thompson’s *I* ^2^ statistics [47]. As the study number was low (< 10), we could not assess the publication bias or additional influence analysis (e.g., leave-one-out analyses).

Forest plots were used to summarize the results graphically. Individual study confidence intervals were presented on the plot using *t*-distribution estimation. We report the results as (MD, [95% CI lower limit – 95% CI upper limit]).

### Risk of bias assessment

Risk-of-bias assessment was performed by two independent authors using the revised Cochrane risk-of-bias tool (RoB2) [48]. Disagreements were solved by consensus. Domains assessed biases resulting from the randomization process, deviations from the intended intervention, missing data, the measurement of the outcome, and the selection of the reported results. The investigators rated each domain, and the risk level was automatically calculated by the algorithm, which could be characterized as low, some concerns, or high.

### Certainty assessment

The Grading of Recommendations, Assessment, Development and Evaluation (GRADE) tool was applied by two independent investigators to assess the quality of the best available evidence [49]. Disagreements were resolved by consensus.

## Results

### Study selection

**Fig. 1:**
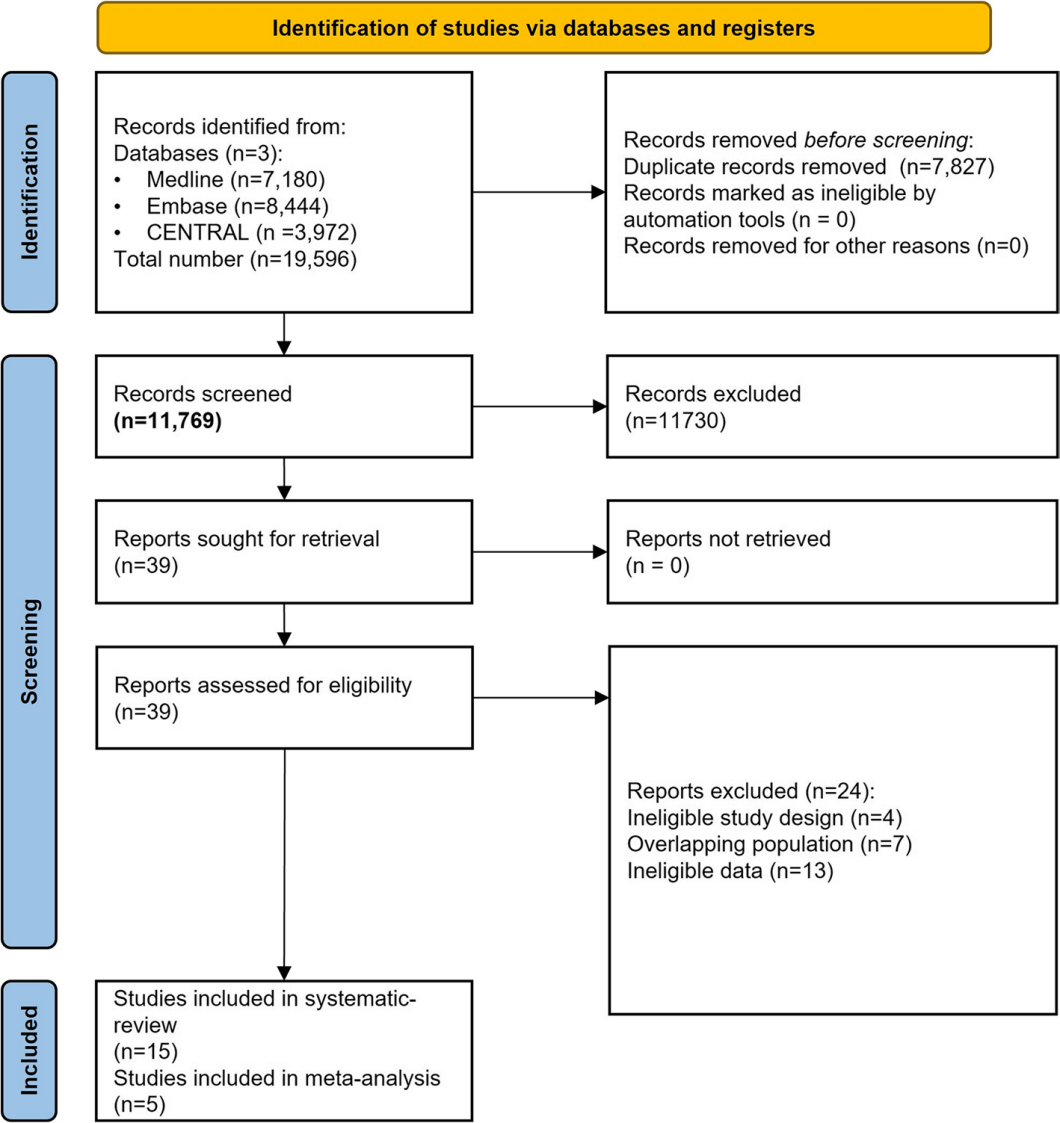
PRISMA flowchart of the selection process

The results of the search and selection processes are summarized in Fig. 1. Our search key identified 19,596 records. Cohen’s kappa index for the title and abstract selection was 0.86, whereas it was 0.95 for the full-text selection. Of the 15 articles eligible for the qualitative synthesis (877 patients), five were suitable for the quantitative synthesis of the Shannon diversity index (335 patients) [43, 50–53] and three for the quantitative synthesis of Chao1 and observed OTUs indices (236 patients) [43, 50, 52]. No additional articles were found by screening the reference lists of the included papers. We included only non-overlapping populations in our review. Most of the studies investigated adult populations. One article investigated neonates [54], and one study included an adolescent population aged 15 years [50]. In eight of the studies, the indication of antibiotic therapy was *Heliobacter pylori* eradication [43, 50, 52, 55–59]. One study focused on *Clostridioides difficile* infection [53], and two investigated patients with various infections outside the gastrointestinal tract [54, 60]. Four studies investigated healthy populations without any medical indication for antibiotic therapy [51, 61–63]. For the investigation of the microbial composition, nine studies used the 16S rRNA sequencing technique [43, 50–56, 61], three used standard microbiological culturing techniques [57–59], one study combined DNA-based terminal restriction fragment length polymorphism (TRFLP) analysis and standard culturing methods [62], and two studies used other polymerase chain reaction (PCR)-based techniques [60, 63]. All included articles were available in full text and were published in peer-reviewed journals, except the study by Amarri et al. [60], which was available as a report on the EU Clinical Trials Register website.

### Study characteristics (Table 1)

**Table 1:** The main characteristics of the included studies

**Study**	**Country**	**Study design** ^a^	**Population**		**Antibiotic (and additional) treatment**			**Probiotic supplementation**		**Microbiome analysis method**
			**Number of randomized patients (female %)**	**Age (years - mean ± SD) in the intervention (and control) groups**	**Indication**	**Type and dose**	**Duration (days)**	**Type and dose**	**Duration (days)**	
Cárdenas et al. (2020) [55]	Ecuador	single-blinded RCT	38 (60.5)	37.9 ± 7.2 (39.5 ± 10.7)	*Helicobacter pylori* infection	Amoxicillin 1 g tid, tinidazole 1 g qid, omeprazole 40 mg bid	14	*Saccharomyces boulardii CNCM I-745* 22.5 × 10^9^ CFU/day	14	16S rRNA sequencing
Chen et al. (2018) [56]	China	open-label RCT	70 (78.5)	43.89 ± 12.50 (43.20 ± 12.45)	*Helicobacter pylori* infection	Pantoprazole 40 mg, amoxicillin 1000 mg, furazolidone 100 mg, colloidal bismuth pectin 0.4 g, bid	14	*Clostridium butyricum* 3 × 40 mg/day	14	16S rRNA sequencing
De Wolfe et al. (2018) [53]	USA	double-blinded, placebo controlled RCT	31 (N.D.)	N.D. (N.D.)	*Clostridioides difficile* infection	Standard of care antibiotics (*vancomycin, metronidazole, or fidaxomicin*)	28	*Lactobacillus acidophilus NCFM® (ATCC 700396), Lactobacillus paracasei Lpc-37 (ATCC SD5275), Bifidobacterium lactis Bi-07 (ATCC SC5220), and Bifidobacterium lactis Bl-04 (ATCC SD5219).* 1.7 × 10^10^ CFU/day	28	16S rRNA sequencing
Kabbani et al. (2017) [61]	USA	open-label RCT	24 (59)	N.D. (N.D.)	Healthy volunteers no indication	Amoxicillin-clavulanate 875/125 mg, bid	7	*Saccharomyces boulardii* (SB) CNCM I-745 (syn. CBS 5926) 2 × 500 mg/day	14	16S rRNA gene pyrosequencing (bTEFAP).
Kakiuchi et al. (2020) [50]	Japan	open-label RCT	65 (44.6)	15.31 ± 0.32 (15.08 ± 0.28)	*Helicobacter pylori* infection	Vonoprazan 20 mg, amoxicillin 750 mg, clarithromycin 400 mg bid	7	*Enterococcus faecium 129 BIO 3B-R.* 3 tablets/day	7	16S rDNA sequencing
MacPherson et al. (2018) [51]	Canada	double-blinded, placebo-controlled RCT	70 (N.D.)	N.D. (N.D.)	Healthy volunteers no indication	Amoxicillin trihydrate 875 mg, potassium clavulanate 125 mg	7	*Lactobacillus rhamnosus* R0011 and *Lactobacillus helveticus* R0052 3.8 × 10^9^ CFU and 0.2 × 10^9^ CFU/day	14	16S rRNA gene amplicon, shotgun metagenomics sequencing
Oh et al. (2016) [43]	Korea	RCT	20 (30)	51.7 ± 0.79 (49.3 ± 3.56)	*Helicobacter pylori* infection	Clarithromycin 500 mg, amoxicillin 1000 mg, lansoprazole 30 mg bid	14	*Streptococcus faecium* and *Bacillus subtili*s 2 × (9 × 10^8^ and 1 × 10^8^)/day	14	16S rRNA gene-pyrosequencing
Tang et al. (2021) [52]	China	placebo-controlled, multi-center RCT	151 (34.4)	43.29 ± 11.30 (45.32 ± 10.98)	*Helicobacter pylori* infection	Esomeprazole 20 mg, amoxicillin 1000 mg furazolidone 100 mg, bismuth potassium citrate 220 mg bid	14	*Enterococcus faecium* and *Bacillus subtilis* 3 × (4.5 × 10^8^ and 5.0 × 10^7^) CFU/day	28	16S rRNA high-throughput sequencing
Zhong et al. (2021) [54]	China	open-label parallel RCT	42 (52.4)	all neonates (all neonates)	15 neonates with neonatal pneumonia 5 neonates with urinary tract infection 35 neonates with non-specific infection	Piperacillin–tazobactam 100 mg/kg bid	7	*Bifidobacterium* *longum, Lactobacillus acidophilus,* and *Enterococcus faecali*s 3 × 1.0 × 10^7^ CFU/day	7	High-throughput sequencing of 16S rRNA amplicons
Engelbrektson et al. (2009) [62]	USA	placebo-controlledRCT	40 (77.5)	36.5 ± N.D. (39.5 ± N.D.)	Healthy volunteers – no indication	Augmentin (amoxicillin and clavulanic acid) 875 mg bid	7	*Bifidobacterium lacti*s Bl-04 (5 × 10^9^ CFU*), Bifidobacterium lactis* Bi-07 (5 × 10^9^ CFU), *Lactobacillus acidophilu*s NCFM (5 × 10^9^ CFU) *Lactobacillus paracasei* Lpc-37 (5 × 10^9^ CFU) and *Bifidobacterium bifidum* Bb-02 (5 × 10^8^ CFU) 2 × 2.05 × 10^10^ CFU/day	21	DNA-based TRFLP analysis and culture-based microbiological techniques
Forssten et al. (2014) [63]	Finland	double-blinded, parallel RCT	80 (50)	33.7 ± 9.4 (30.9 ± 10.3)	Healthy volunteers – no indication	Amoxicillin 875 mg, clavulanate 125 mg	7	*Lactobacillus acidophilus (L. acidophilus) ATCC 700396 and Bifidobacterium animalis (B. animalis) ssp. Lactis ATCC SD5220 (Danisco)* 12.5 × 10^9^ and 12.5 × 10^9^ CFU/day	14	qPCR and flow cytometry
Madden et al. (2005) [57]	UK	pilot-scale, double-blinded RCT	13 (53.8)	60 ± N.D. (49 ± N.D.)	*Helicobacter pylori* infection	Amoxycillin 500 mg qid, metronidazole 400 mg tid, lansoprazole 30 mg bid	8	*Lactobacillus acidophilus (CLT60 and CUL21) and two strains of Bifidobacterium bifidum (CUL17 and B. bifidum Rhodia* 2.5 × 10^10^ CFU/day	14	Culture-based microbiological techniques
Plummer et al. (2005) [58]	UK	double-blinded RCT	155 (N.D.)	N.D. N.D.	*Helicobacter pylori* infection	Amoxicillin 1 g bid, clarithromycin 500 mg bid, lansoprazole 30 mg bid; in case of penicillin allergy, metronidazole 400 mg tid was substituted	7	*Lactobacillus acidophilus* (CUL60 and CUL21) and two strains of *Bifidobacterium spp* 2.5 × 10^10^ CFU/day	21	Culture-based microbiological techniques
Wang et al. (2017) [59]	China	double-blinded RCT	20 (45)	37.1 ± 12.3 (42.8 ± 13.8)	*Helicobacter pylori* infection	Esomeprazole 20 mg bid, amoxicillin 1000 mg bid, clarithromycin 500 mg bid, tinidazole 500 mg bid	14	*Saccharomyces* *boulardii* CNCM I-745® 2 × 500 mg	14	Culture-based microbiological techniques
Amarri et al. (2008) [60]	Italy	open-label, national, parallel RCT	58 (50)	40 ± 18.9 months (42.1 ± 18.9 months)	Bacterial upper respiratory tract infections	Amoxicillin 50 mg/kg/day divided in 3 daily doses	5-10	Antibiotic-resistant *Bacillus* *clausii* 2 × 2 × 10^9^ CFU/day	12-17	PCR-DGGE

*Abbreviations*: *RCT* Randomized controlled trial, *USA* United States of America, *UK* United Kingdom, *N.D*. No data, *bid* Twice a day; *tid*, three times a day, *qid* Four times a day, *bTEFAP* Bacterial tag–encoded FLX amplicon pyrosequencing, *DNA* Deoxyribonucleic acid, *TRFLP* Terminal restriction fragment length polymorphism, *qPCR* Quantitative real-time polymerase chain reaction *PCR-DGGE* Polymerase chain reaction denaturing gradient gel electrophoresis, *rRNA* Ribosomal ribonucleic acid, *CFU* Colony forming unit

^a^If not otherwise mentioned, the studies were single centers

#### The impact of probiotic supplementation during antibiotic therapy on the Shannon diversity index

We identified eight eligible articles reporting the results of the Shannon diversity index [43, 50–56], but only six provided the data (in numerical or boxplot form) for meta-analysis [43, 50–54]. The article that reported on the neonate population exclusively [54] was not included in the meta-analysis due to the impact on the indirectness of our results [18].

**Fig. 2:**
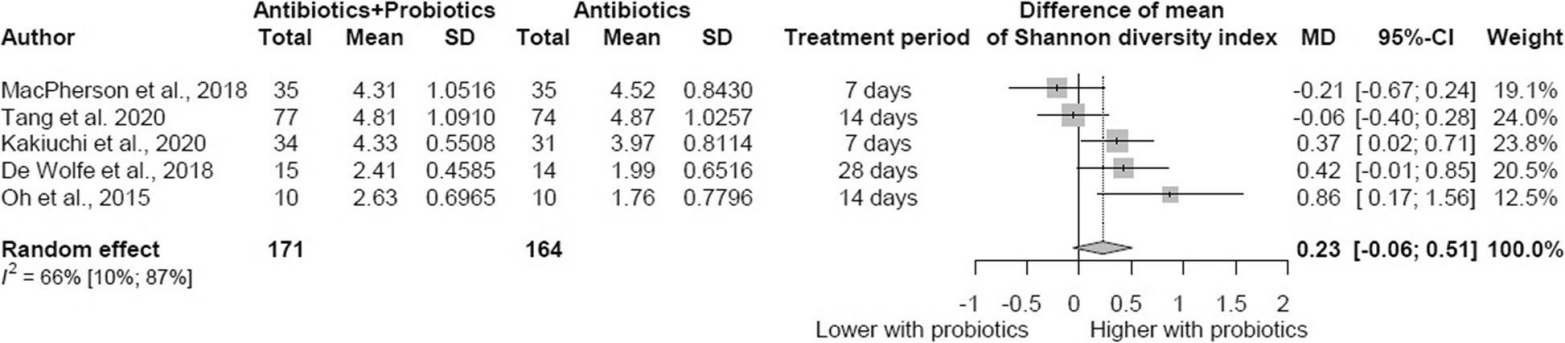
After antibiotic treatment, the Shannon diversity index is not significantly higher in patients receiving concurrent probiotic supplementation than in those treated with antibiotics alone, as measured immediately after antibiotic treatment. CI, confidence interval; MD, mean difference

The results of the meta-analysis including five articles with 335 patients are summarized in Fig. 2. On the basis of our results, the gut microbiome diversity was not significantly different between the probiotic-supplemented and antibiotic-only treated groups when measured immediately at the end of antibiotic treatment. The mean difference in Shannon diversity index between the intervention and control groups was 0.23 [(−)0.06 – 0.51].

Although all the included studies were RCTs, the baseline values in the article by De Wolfe et al. [53] showed a marked difference between the probiotic and control groups (MD = 0.64 [(0.05*–*1.22]). As a sensitivity analysis, we performed a separate calculation for data before the treatment and for the “before-after” values change in each study (Additional File 3: Fig. S1-2). We did not find any significant difference in the change values between the experimental and control groups regarding the Shannon diversity index (MD = 0.07 [(−)0.19*–*0.32]).

#### The impact of probiotic supplementation during antibiotic therapy on the Chao1 index

**Fig. 3:**
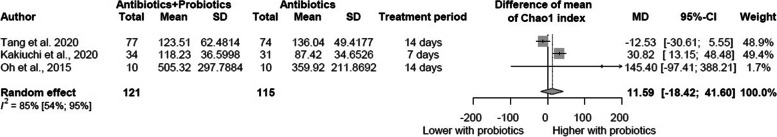
The Chao1 index is not significantly higher in the group receiving concurrent probiotic supplementation than in the group treated with antibiotics alone as measured immediately after antibiotic treatment. CI, confidence interval; MD, mean difference

We identified three eligible articles with 236 patients in total for the meta-analysis of Chao1 index, [43, 50, 52]. The results are presented in Fig. 3. The results of Kabbani et al. were previously excluded due to the time point of measurement, which was not reported precisely [61]. According to our results, the mean difference of Chao1 index between the intervention and control groups was 11.59 [(−)18.42–41.60], meaning that the diversity of the intestinal flora of the two groups did not significantly differ from each other.

As there was a large difference in the baseline values between the intervention and control groups in the article of Kakiuchi et al. [50] (MD = 21.57 [3.47–39.68]), here, we also performed an additional sensitivity analysis for the baseline values and the changes (Additional File 3: Fig. S3-4) with no significant difference in the latter between the groups (MD = 3.77 [(−)10.17–17.71]).

#### The impact of probiotic supplementation during antibiotic therapy on observed OTUs

**Fig. 4:**
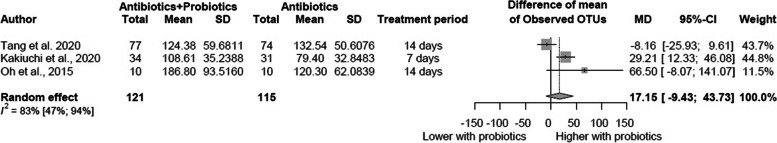
The number of Observed OTUs is not significantly higher in the group receiving concurrent probiotic supplementation than in the group treated with antibiotics alone, as measured immediately after antibiotic treatment. OTU, operational taxonomic unit; CI, confidence interval; MD, mean difference

Three of the six articles reporting on Observed OTUs were eligible for quantitative analysis [43, 50, 52]. Others were excluded due to qualitative data reporting [55], not precisely defined time point of measurement [61], or due to the age of the population (neonates) [54]. Results are presented in Fig. 4. According to our results, probiotic supplementation did not result in a significantly different microbiome diversity compared to the antibiotic-only treated group. The mean difference of observed OTUs between the intervention and control groups was 17.15 [(−)9.43–43.73].

The additional sensitivity analysis for the baseline and the change values revealed no significant difference between groups either (Additional File 3: Fig. S5-6) (change: MD = 8.09 [(−)3.87–20.05]).

### Qualitative synthesis

#### The impact of simultaneous probiotic supplementation during antibiotic treatment on α-diversity indices

The results of α-diversity indices of the studies, adding those that were not included in the meta-analysis, are summarized in Table 2. The α-diversity indices were lower after the antibiotic administration in both the intervention and the control groups. The three articles — that were not included in the meta-analysis — reporting on the Shannon diversity index revealed no significant difference between the groups [54, 55, 64]. As for the observed OTUs, the three articles not included in the meta-analysis reported no significant difference between the two groups [54, 55, 61]. Regarding the Chao1 index, Kabbani et al. reported significantly higher values in the control group [61]. For most of the α-diversity indices that were not suitable for meta-analysis, the studies did not reveal a significant difference (5% significance level) between the probiotic and control groups. Overall, from the nine studies reporting on α-diversity indices, three were able to show a significant effect of probiotics on at least one index [43, 50, 55]. However, we did not find any common but distinguishable aspects that could explain the similar results.

**Table 2:**
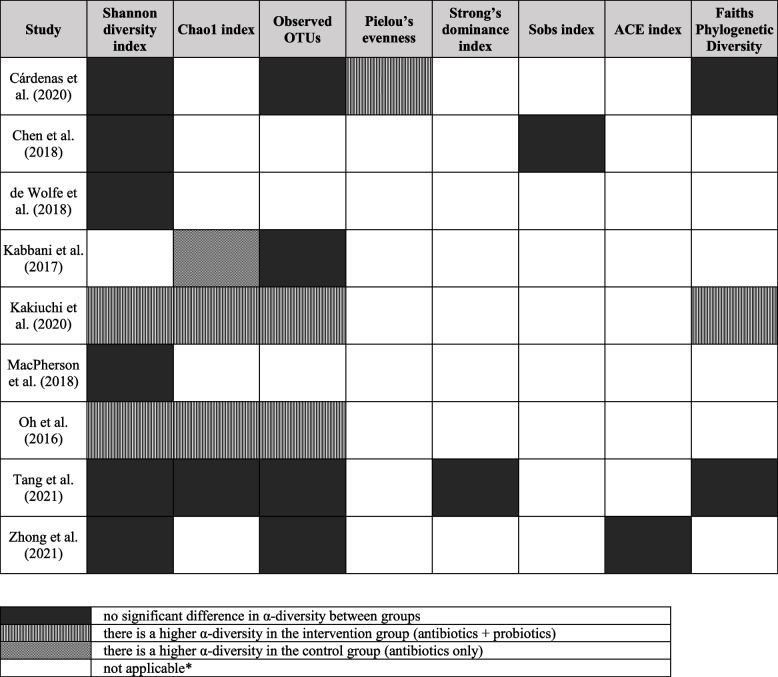
Changes in the microbiome α-diversity indices as measured after the antibiotic treatment

Definitions for each outcome are detailed in Additional File 2: Table S2 [20–36]

^*^If a study did not investigate a specific outcome, “not applicable” is indicated

*Abbreviations*: *OTU* Operational taxonomic unit, *ACE* Abundance-based coverage estimator

#### The impact of simultaneous probiotic supplementation during antibiotic treatment on β-diversity indices

The summarized results of ß-diversity indices are presented in Table 3. The most used ß-diversity indices were Bray-Curtis dissimilarity index and both weighted and unweighted UniFrac (unique fraction metric) distances. Most studies found no significant difference (5% significance level) between the groups. Only Engelbrektson et al. reported a significantly improved ß-diversity by the Euclidean distance [62] in the intervention group. After antibiotic therapy, almost no change occurred in the probiotic group, while there was a large shift toward diminished ß-diversity in the control group. None of the studies reported significant differences between the two groups regarding other indices [50–53, 55].

**Table 3:**
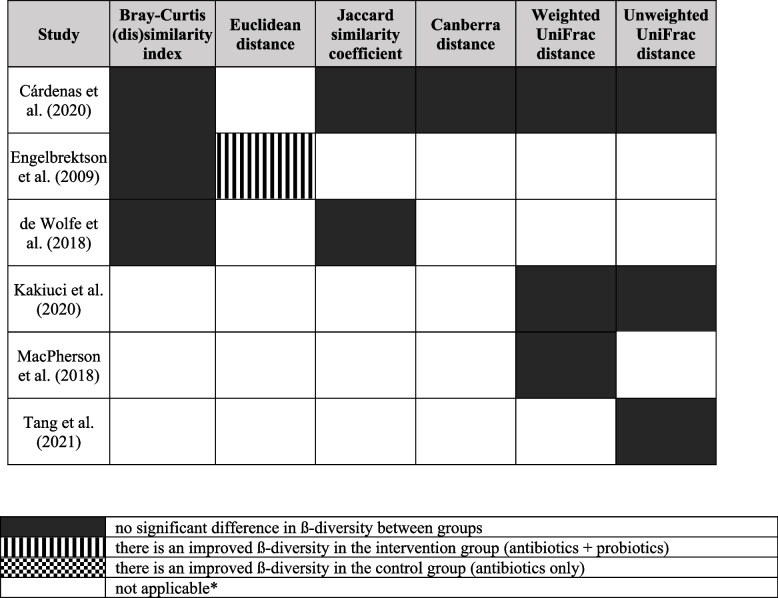
The systematic review of the microbiome ß-diversity indices as measured immediately after the completion of antibiotic treatment

Definitions for each outcome are detailed in Additional File 2: Table S2 [20–36]

^*^If a study did not investigate a specific outcome, “not applicable” is indicated

*Abbreviations*: *UniFrac* Unique fraction metric

### Taxonomic analysis of microbiome composition

At phylum level, a decreasing trend in the proportion of *Firmicutes* and *Bacteroidetes* with a higher relative abundance of *Proteobacteria* was observed after antibiotic therapy in both groups, regardless of probiotic supplementation. There was also a reduction in the *Bacteroidetes:Firmicutes* (B:F) ratio at the cessation of treatments. This was confirmed by several studies; however, in the study of Oh et al., the reduction was significantly greater in the control group [43, 52, 56]. Importantly, these changes in phyla abundances disappeared at day 56 in the studies of Chen et al. and Tang et al. [52, 64].

Changes in the level of *Enterobacteriaceae* family were inconsistent across the studies. Several articles reported an increasing trend of *Enterobacteriaceae* in the probiotic supplemented group only [55, 62]; however, according to other studies [57, 58], this increase was observed only in the control group. Meanwhile, Forssten et al. and MacPherson et al. reported a higher relative abundance of *Enterobacteriaceae* in both groups after antibiotic treatment, which normalized after 2 weeks of follow-up [51, 63]. Changes in the other bacterial families were heterogeneously reported (see Tables S3 and S4).

At genus level, *Bacteroides* showed a decreasing trend in the probiotic supplemented group [53, 55, 57]. However, some studies reported a reduction of *Bacteroides* in both groups after antibiotic treatment, which showed a re-growing tendency during 3–8 weeks of follow-up [52, 58]. Patients with probiotic supplementation had a higher proportion of *Escherichia spp*. according to Cárdenas et al. [55], while two other studies reported that the addition of probiotics reduced the overgrowth of *Escherichia* compared to the control group [43, 61]. According to the study of Tang et al., where probiotic supplementation was continued for two more weeks after antibiotics cessation, the abundance of genus *Enterococcus* increased at weeks 2 and 4 of follow-up in the intervention group only [52]. Meanwhile, Wang et al. reported this increasing tendency in both groups at week 2. In their study, probiotics were suspended after the antibiotic cessation. However, by weeks 6, 8, and 9 of follow-up, the enrichment of *Enterococcus* had disappeared in both intervention and control groups as reported in both of the studies [52, 59]. Probiotic supplementation seems to help maintain the level of *Bifidobacterium* genus [50, 54, 62]. According to Plummer et al., *Bifidobacterium* decreased in both groups during antibiotic therapy but tended to increase after therapy cessation to day 35 of follow-up [58]. In the study of Kabbani et al., *Roseburia* prevalence was decreased by antibiotic treatment only; however, Tang et al. reported a significant reduction in both groups [52, 61]. Probiotic supplementation resulted either in an increase of *Blautia* in the intervention group or decrease in the control group only according to two studies [50, 54]. However, Tang et al. described a lower abundance of *Blautia* in both groups after antibiotic treatment, with a re-growing tendency with time regardless of probiotic supplementation [52].

The summarized results of the taxonomic analysis of microbiome composition, as measured immediately at the end of simultaneous antibiotic and probiotic treatment, are presented in Additional File 2: Table S3. The results of the follow-up measurements (after cessation of antibiotic and probiotic treatments) are summarized in Additional File 2: Table S4.

### Risk of bias assessment

The results of the risk of bias assessment are detailed in Additional File 2: Tables S5-6 and Additional File 3: Fig. S7-12. The overall risk of bias was low to high for the indices included in the meta-analyses. The high risk of bias was caused mainly by the baseline differences between interventional and control groups regarding some of the diversity indices [50, 53].

On the basis of the GRADE assessment, the quality of evidence for the meta-analyses was low (Additional File 2: Table S7).

## Discussion

This study is the first systematic review and meta-analysis that summarizes the results of the currently available randomized controlled trials investigating the effect of probiotic supplementation during antibiotic treatment on the gut microbiome. Probiotic supplementation to prevent antibiotic-induced dysbiosis is not supported by our results.

The imbalance of the bacterial composition in the gut microbiome is called dysbiosis. One form of this can be low-diversity dysbiosis, which is often caused by broad-spectrum antibiotic therapy [1]. Decreased gut microbiome diversity has been associated with obesity, inflammatory bowel disease, liver disease, and recurrent *Clostridioides difficile* infection, among other pathologies. Maintaining the gut microbial diversity during periods of potential impairment seems important [3, 65]. Probiotics are widely used to prevent this dysbiotic state during antibiotic therapy; however, their role and effect on the gut microbiome are still in question.

The results of our systematic review and meta-analysis do not support probiotic supplementation during antibiotic therapy in order to prevent low-diversity dysbiosis. The meta-analysis of Shannon, Chao1, and observed OTUs diversity indices did not show a significant effect of probiotics on maintaining diversity. According to the current evidence, a single index is insufficient to describe bacterial communities [13–15]. Our quantitative results of three alpha diversity indices indicate a lack of significant effect of probiotic supplementation on gut microbiome diversity during antibiotic therapy. We could not include many of the identified reported data in the quantitative analysis as several studies provided only narrative results. However, these data confirm the findings of the meta-analyses as most studies concluded that there were no significant differences in diversity between the probiotic supplemented and the antibiotics alone groups after antibiotic therapy. As for other indices describing α- and ß-diversity, most studies found no significant difference between the two groups, especially when comparing ß-diversities. In conclusion, according to currently available data, there is no evidence that probiotic supplementation has a relevant effect on gut bacterial diversity during antibiotic therapy.

Antibiotic-induced changes in the gut microbial communities, such as decreased *Bacteroidetes:Firmicutes* (B:F) ratio has been associated with obesity and the metabolic syndrome [66, 67]. This tendency of reduction in the B:F ratio was observed regardless of probiotic supplementation during antibiotic therapy according to several included studies, but it also normalizes in both groups during follow-up [52, 64]. Increased proportion of *Proteobacteria* was reported by studies in both groups, which is a possible microbial signature of several diseases, such as metabolic disorders and inflammatory bowel disease [68]. These changes, however, showed a restoration tendency after 8 weeks of follow-up [43, 52, 56].

The enrichment of the *Enterobacteriaceae* family is commonly associated with specific antibiotic resistance genes for aminoglycosides, beta-lactams, and carbapenems, thus being a potentially dangerous source of antibiotic resistance gene transfer [51, 69]. Although the members of this family are considered normal intestinal residents, some may become opportunistic pathogens. They have a higher abundance in inflammatory bowel disease patients, but their underlying pathological mechanisms are still under investigation [70]. Changes in the level of *Enterobacteriaceae* family were inconsistently reported in the included articles; therefore, we cannot draw strong conclusions about the consequences of probiotic supplementation. The tendency of abundance normalization after the cessation of the antibiotic treatment suggests that the changes in *Enterobacteriaceae* induced by treatment are transient [51, 63].

Reduced abundance of several species in *Bacteroides* might be associated with the risk of *Clostridioides difficile* infection [71]. The reduction of this genus was prevalent in the probiotic supplemented group in several cases, the background of which is unclear [53, 55, 57]. The re-growth of these bacteria during follow-up suggests that the changes are not permanent [52, 58].

*Escherichia coli* and *Enterococcus* family species are commensal inhabitants of the gastrointestinal tract that may become pathogens in a dysbiotic environment for several diseases, such as antibiotic-associated diarrhea, vomiting, or permanent intestinal inflammation. Moreover, they are characterized by antibiotic resistance [72, 73]. Probiotic supplementation seems to reduce *Escherichia* overgrowth during antibiotic therapy according to Kabbani et al. and Oh et al. [43, 61]. Nevertheless, the level of both *Escherichia* and *Enterococcus* tends to normalize after antibiotics cessation regardless of probiotics supplementation. This brings the efficacy of probiotics in preventing this type of antibiotic induced dysbiosis into question [52, 59, 61].

Probiotic supplementation seems to maintain the level of *Bifidobacteria* during antibiotic therapy [50, 54, 62]. Several species and/or strains of this genus may be useful for health, including modulating gut microbial homeostasis, inhibiting pathogens, and modulating immune responses. They can suppress the oncogenic activity within the microbiome, and they are able to produce vitamins and transform food compounds into bioactive molecules [74]. *Bifidobacteria* also play a crucial role during early life. They are among the first colonizers of the human gut. According to previous studies, children with allergic diseases have a reduced gut microbial diversity with lower abundance of *Bifidobacterium*, *Lactobacillus*, and *Bacteroides* compared to healthy controls [75]. Therefore, the results of Zhong et al. are especially important as they showed that probiotic supplementation was able to maintain the level of *Bifidobacteria* in newborns during antibiotic therapy [54].

Some of the included articles suggested that probiotic supplementation during antibiotic therapy has a protective effect on *Blautia* and *Roseburia spp*. levels [50, 54, 61]. Recently, *Blautia* has been associated with the alleviation of inflammatory and metabolic diseases by regulating host health, and it has also been characterized by antibacterial activity [76]. Gut *Roseburia spp.* produce short-chain fatty acids, modulate colonic motility, support immunity, and have anti-inflammatory effects [77]. These findings suggest that probiotic supplementation may have some benefits but the tendency for *Blautia* levels to normalize spontaneously after antibiotic discontinuation casts doubt on them [52].

### Implication for practice and research

The summary of the available literature facilitates the utilization of scientific results in daily practice, which is crucially important [78, 79]. According to our findings, probiotics have only a minimal and temporary effect on the composition and diversity of gut microbiome during antibiotic therapy and are not suitable for preventing antibiotic-induced low-diversity dysbiosis. In this regard, strain-specific probiotic supplementation with antibiotics may be considered especially for vulnerable groups to prevent *Clostridioides difficile* infection or antibiotic-associated diarrhea, as advised by the current guideline of the AGA and WGO on the use of probiotics [11, 12]. These findings were however not connected to gut microbial compositions. Given our results, which describe a low moderating effect on gut flora, the question arises as to what exactly is the mechanism by which probiotics help prevent these conditions. A recent meta-analysis also points out that some strains may be more effective in the prevention of diarrhea and that the effect depends on the initial risk level. According to this, patients with a low baseline diarrhea risk do not benefit from probiotic supplementation during antibiotic treatment [80]. Our findings do not suggest any further benefit regarding microbiome composition. Further evaluation of the relation between clinical manifestations, microbial diversity indices, and taxonomic composition will bring a more comprehensive understanding of the role of gut microbes in human health and how different factors affect it. The standardization of methods for microbiome diversity measurement and the definition of its optimal value are key factors in generating more homogenous data with increased clinical relevance. A measurement after a standard follow-up period should be considered for all future similar studies to determine the long-term effects.

The relatively small number of publications and the wide range of methods and diversity indices used across the eligible articles indicate that microbiome diversity is an under-researched area and that professional consensus is still lacking. Although recommendations for the conduct and reporting of microbiome research have been published, there are no standards for the choice of diversity indices [81, 82]. Moreover, every year, new approaches to characterize microbiome composition are emerging, making standardization increasingly difficult [83]. Similarly, the relationship of microbiome composition and its changes with physiological functions and clinical symptoms is not well understood yet: no evident clinical characteristics or symptoms can be attributed to the different diversity index values, especially their numerical variation. Our results suggest that the routine use of probiotics is not justified for maintaining gut microbial balance and diversity. This is particularly important for outpatients who are at low risk and usually start taking probiotics for this purpose. This finding is in accordance with the current AGA recommendation, which also highlights that patients with low risk would reasonably select no probiotics, thus avoiding potential harm and additional costs [12]. In order to determine the exact role of probiotics in the clinic, these questions need to be answered through further professional discussion and intensive research.

### Strengths and limitations

The main strength of the study is the high level of evidence for our quantitative results as we included only randomized controlled trials in our review and meta-analysis. Moreover, to our knowledge, this is the first meta-analysis and the most comprehensive review of the topic to date. We followed the strict guidelines of Cochrane recommendations [18] and PRISMA Statement [19] when performing our systematic review and meta-analysis, which is strengthening our results.

Although we have identified all relevant studies published on the topic without setting restrictions on microbial variables, we acknowledge the limited availability of eligible articles. Our strict inclusion criterion of using only results from randomized controlled trials ensured the highest level of evidence, despite potentially reduced the number of included articles and cases. This emphasizes the necessity for further high-quality studies. Due to the small sample sizes and the limited number of studies, the results of the meta-analysis of the investigated diversity indices should be handled with criticism. We could not perform a quantitative synthesis of much of the data either due to insufficient reporting and high variability regarding the methods and indices used to measure and describe the gut microbial composition and diversity. ASVs (amplicon sequence variant) are generally considered more accurate method to represent groups of DNA sequences than OTUs as they do not rely on arbitrary similarity thresholds and can identify individual variants within a taxon. However, due to the lack of available studies meeting inclusion criteria using ASV analysis, our meta-analysis utilized OTU data, which did not however hinder the interpretation of consistent results obtained from before-and-after comparisons. Factors such as the use of different bacterial strains as probiotics, varied type and dose of antibiotics, inclusion of subjects with different health conditions (including both diseased and healthy individuals without infections), as well as variations in age across the included studies, could have contributed to the lack of conclusive evidence regarding efficacy. These factors should be taken into consideration when interpreting the results and highlight the need for further research to better understand the impact of these variables on the outcomes.

## Conclusions

The summarized results of the currently available randomized controlled trials cannot support probiotic supplementation during antibiotic therapy to prevent low-diversity dysbiosis. The meta-analyses of Shannon, Chao1, and observed OTUs diversity indices showed no significant effect of probiotics on maintaining diversity. Although we could not analyze all the identified results quantitatively, a tendency of no modulating effect of probiotics was observed for other reported α- and β-diversity indices as well. Changes in the taxonomic composition tend to be similar in the intervention and control groups; however, it varies between the different studies The tendency of microbiome restoration after a 3–8-week follow-up period, regardless of probiotic supplementation and remission of the differences between the intervention and control groups, challenges the questions on the benefits of routine probiotic supplementation during antibiotic treatment. There is a strong need to standardize methods and indicators, to build professional consensus, and to continue intensive research on clinical relevance.

## Supplementary Information

**Additional file 1:** **Supplementary Methods S1.** Details of the study protocol; **Supplementary Methods S2.** Details of the systematic search.

**Additional file 2:** **Table S1.** PRISMA checklist 2020; **Table S2.** Definitions of gut microbiome diversity outcomes reported in the included studies; **Table S3.** The summarized results of taxonomic analysis of microbiome composition as measured immediately at the end of simultaneous antibiotic and probiotic treatment; **Table S4.** Outcomes of follow-up as reported in each study; **Table S5.** Risk of bias assessment for all outcomes - Assignment to intervention; **Table S6.** Risk of bias assessment for all outcomes - Adhering to intervention; **Table S7.** GRADE assessment for the meta-analyses of Shannon, Chao1 and Observed OTUs diversity indices.

**Additional file 3:** **Fig. S1.** Additional sensitivity analysis for the baseline values of Shannon diversity index; **Fig. S2.** Additional sensitivity analysis for the change between the “before-after” values of Shannon diversity index; **Fig. S3.** Additional sensitivity analysis for the baseline values of Chao1 index; **Fig. S4.** Additional sensitivity analysis for the change between the “before-after” values of Chao1 index; **Fig. S5.** Additional sensitivity analysis for the baseline values of Observed OTUs; **Fig. S6.** Additional sensitivity analysis for the change between the “before-after” values of Observed OTUs; **Fig. S7.** Risk of bias assessment for the main meta-analysis of Shannon diversity index - Assignment to intervention; **Fig. S8.** Risk of bias assessment for the main meta-analysis of Shannon diversity index - Adhering to intervention ; **Fig. S9.** Risk of bias assessment for the meta-analysis of Chao1 index - Assignment to intervention; **Fig. S10.** Risk of bias assessment for the meta-analysis of Chao1 index - Adhering to intervention**Fig. S11.** Risk of bias assessment for the meta-analysis of Observed OTUs - Assignment to intervention; **Fig. S12.** Risk of bias assessment for the meta-analysis of Observed OTUs - Adhering to intervention.

## Data Availability

The datasets used and analyzed during the current study are available from the corresponding author on reasonable request. The original datasets used in this study can be found in the full-text publications included in the systematic review and meta-analysis.

## Abbreviations

16S rRNA: 16S ribosomal ribonucleic acid
ACE: Abundance-based coverage estimator
AGA: American Gastroenterological Association
ASV: Amplicon sequence variant
B:F ratio: *Bacteroidetes:Firmicutes* ratio
CFU: Colony forming unit
bid: Twice a day
CI: Confidence interval
DNA: Deoxyribonucleic acid
GRADE: The Grading of Recommendations, Assessment, Development and Evaluation
MD: Mean difference
OTU: Operational taxonomic unit
PCR: Polymerase chain reaction
PCR-DGGE: Polymerase chain reaction denaturing gradient gel electrophoresis
PRISMA: Preferred Reporting Items for Systematic Reviews and Meta-Analyses
PROSPERO: International prospective register of systematic reviews
RCT: Randomized controlled trial
RoB: Risk of bias
tid: Three times a day
TRFLP: Terminal restriction fragment length polymorphism
UK: United Kingdom
UniFrac: Unique fraction metric
USA: United States of America
qid: Four times a day
qPCR: Quantitative real-time polymerase chain reaction
WGO: World Gastroenterology Organization
