# Supplementary material for: Probiotic supplementation during antibiotic treatment is unjustified in maintaining the gut microbiome diversity: a systematic review and meta-analysis
Source: BMC Med. 2023 Jul 19;21:262. doi: 10.1186/s12916-023-02961-0 (PMC10355080; doi:10.1186/s12916-023-02961-0)
Supplement: Supplementary file 1 — Additional file 1: Supplementary Methods S1. Details of the study protocol; Supplementary Methods S2. Details of the systematic search. [file 12916_2023_2961_MOESM1_ESM.docx]

**Supplementary Methods S1**

We previously submitted our study protocol to the **International prospective register of systematic reviews (PROSPERO) (CRD42021282983)**.

The study protocol can be accessed via the following link:

<https://www.crd.york.ac.uk/prospero/display_record.php?RecordID=282983>

**Supplementary Methods S2 – Details of the systematic search**

| **Date of search: October 15, 2021** | |
| --- | --- |
| **Databases** | |
|  | MEDLINE (via PubMed) (7180),  Embase (8444),  Cochrane Central Register of Controlled Trials (CENTRAL) (3972) |
| **Search key** | |
|  | (probiotic OR probiotic* OR bifidobac* OR lactobac* OR escherichia OR streptococcus OR saccharomyces OR bacillus OR pediococc* OR leuconostoc OR enterococc* OR lactococc*)  AND  ((microbio* OR microbiome OR flora OR microflora)  AND  (diversity OR composition OR abundance OR alteration OR restoration OR reconstitution OR recovery OR correction OR correct* OR disrupt*)) OR (OTU or OTUs OR "operational taxonomic unit*" OR dysbiosis OR dysbacteriosis OR "16S rRNA" OR "rRNA, 16S" OR "16S Ribosomal RNA" OR "RNA, 16S Ribosomal" OR "Ribosomal RNA, 16S" OR "16S rDNA" OR "rDNA, 16S" OR "16S Ribosomal DNA" OR "DNA, 16S Ribosomal" OR "Ribosomal DNA, 16S")  AND  random* |
| **Search key expansions** | |
| **MEDLINE via PubMed** | |
|  | ((("probiotic s"[All Fields] OR "probiotical"[All Fields] OR "probiotics"[MeSH Terms] OR "probiotics"[All Fields] OR "probiotic"[All Fields] OR "probiotic*"[All Fields] OR "bifidobac*"[All Fields] OR "lactobac*"[All Fields] OR ("escherichia"[MeSH Terms] OR "escherichia"[All Fields] OR "escherichiae"[All Fields]) OR ("streptococcus"[MeSH Terms] OR "streptococcus"[All Fields]) OR ("saccharomyces"[MeSH Terms] OR "saccharomyces"[All Fields] OR "saccharomyce"[All Fields]) OR ("bacillu"[All Fields] OR "bacillus"[MeSH Terms] OR "bacillus"[All Fields]) OR "pediococc*"[All Fields] OR ("leuconostoc"[MeSH Terms] OR "leuconostoc"[All Fields] OR "leuconostocs"[All Fields]) OR "enterococc*"[All Fields] OR "lactococc*"[All Fields]) AND (("microbio*"[All Fields] OR ("microbiome s"[All Fields] OR "microbiomic"[All Fields] OR "microbiomics"[All Fields] OR "microbiota"[MeSH Terms] OR "microbiota"[All Fields] OR "microbiome"[All Fields] OR "microbiomes"[All Fields]) OR ("flora"[All Fields] OR "florae"[All Fields] OR "floras"[All Fields]) OR ("gastrointestinal microbiome"[MeSH Terms] OR ("gastrointestinal"[All Fields] AND "microbiome"[All Fields]) OR "gastrointestinal microbiome"[All Fields] OR "microflora"[All Fields] OR "microflorae"[All Fields] OR "microfloras"[All Fields])) AND ("diverse"[All Fields] OR "diversely"[All Fields] OR "diversities"[All Fields] OR "diversity"[All Fields] OR ("composite"[All Fields] OR "composite s"[All Fields] OR "composited"[All Fields] OR "composites"[All Fields] OR "compositing"[All Fields] OR "composition"[All Fields] OR "compositional"[All Fields] OR "compositions"[All Fields]) OR ("abundance"[All Fields] OR "abundances"[All Fields] OR "abundancies"[All Fields] OR "abundancy"[All Fields] OR "abundant"[All Fields]) OR ("alter"[All Fields] OR "alterated"[All Fields] OR "alteration"[All Fields] OR "alterations"[All Fields] OR "altered"[All Fields] OR "altering"[All Fields] OR "alters"[All Fields]) OR ("restorability"[All Fields] OR "restorable"[All Fields] OR "restorated"[All Fields] OR "restoration"[All Fields] OR "restoration s"[All Fields] OR "restorations"[All Fields] OR "restorative"[All Fields] OR "restoratives"[All Fields] OR "restore"[All Fields] OR "restored"[All Fields] OR "restores"[All Fields] OR "restoring"[All Fields]) OR ("reconstitute"[All Fields] OR "reconstituted"[All Fields] OR "reconstitutes"[All Fields] OR "reconstituting"[All Fields] OR "reconstitution"[All Fields] OR "reconstitutional"[All Fields] OR "reconstitutions"[All Fields] OR "reconstitutive"[All Fields]) OR ("recoveries"[All Fields] OR "recovery"[All Fields]) OR ("correct"[All Fields] OR "correctability"[All Fields] OR "correctable"[All Fields] OR "corrected"[All Fields] OR "correctible"[All Fields] OR "correcting"[All Fields] OR "correction"[All Fields] OR "corrections"[All Fields] OR "corrective"[All Fields] OR "correctives"[All Fields] OR "correctness"[All Fields] OR "corrects"[All Fields]) OR "correct*"[All Fields] OR "disrupt*"[All Fields]))) OR ("OTU"[All Fields] OR "OTUs"[All Fields] OR "operational taxonomic unit*"[All Fields] OR ("dysbiosis"[MeSH Terms] OR "dysbiosis"[All Fields] OR "dysbioses"[All Fields]) OR ("dysbiosis"[MeSH Terms] OR "dysbiosis"[All Fields] OR "dysbacteriosis"[All Fields]) OR "16S rRNA"[All Fields] OR "rrna 16s"[All Fields] OR "16S Ribosomal RNA"[All Fields] OR "rna 16s ribosomal"[All Fields] OR "ribosomal rna 16s"[All Fields] OR "16S rDNA"[All Fields] OR "rdna 16s"[All Fields] OR "16S Ribosomal DNA"[All Fields] OR (("dna"[MeSH Terms] OR "dna"[All Fields]) AND "16s"[All Fields] AND ("ribosome s"[All Fields] OR "ribosomes"[MeSH Terms] OR "ribosomes"[All Fields] OR "ribosomal"[All Fields] OR "ribosome"[All Fields])) OR (("dna, ribosomal"[MeSH Terms] OR ("dna"[All Fields] AND "ribosomal"[All Fields]) OR "ribosomal dna"[All Fields] OR ("ribosomal"[All Fields] AND "dna"[All Fields])) AND "16s"[All Fields]))) AND "random*"[All Fields] |
| **Embase** | |
|  | (('probiotic'/exp OR probiotic OR probiotic* OR bifidobac* OR lactobac* OR 'escherichia'/exp OR escherichia OR 'streptococcus'/exp OR streptococcus OR 'saccharomyces'/exp OR saccharomyces OR 'bacillus'/exp OR bacillus OR pediococc* OR 'leuconostoc'/exp OR leuconostoc OR enterococc* OR lactococc*) AND (microbio* OR 'microbiome'/exp OR microbiome OR 'flora'/exp OR flora OR 'microflora'/exp OR microflora) AND ('diversity'/exp OR diversity OR composition OR 'abundance'/exp OR abundance OR alteration OR 'restoration'/exp OR restoration OR reconstitution OR 'recovery'/exp OR recovery OR 'correction'/exp OR correction OR correct* OR disrupt*) OR otu OR otus OR 'operational taxonomic unit*' OR 'dysbiosis'/exp OR dysbiosis OR 'dysbacteriosis'/exp OR dysbacteriosis OR '16s rrna'/exp OR '16s rrna' OR 'rrna, 16s'/exp OR 'rrna, 16s' OR '16s ribosomal rna'/exp OR '16s ribosomal rna' OR 'rna, 16s ribosomal' OR 'ribosomal rna, 16s'/exp OR 'ribosomal rna, 16s' OR '16s rdna'/exp OR '16s rdna' OR 'rdna, 16s'/exp OR 'rdna, 16s' OR '16s ribosomal dna'/exp OR '16s ribosomal dna' OR 'dna, 16s ribosomal' OR 'ribosomal dna, 16s'/exp OR 'ribosomal dna, 16s') AND random* |
| **Cochrane Central Register of Controlled Trials (CENTRAL)** | |
|  | (probiotic OR probiotic* OR bifidobac* OR lactobac* OR escherichia OR streptococcus OR saccharomyces OR bacillus OR pediococc* OR leuconostoc OR enterococc* OR lactococc*) AND ((microbio* OR microbiome OR flora OR microflora) AND (diversity OR composition OR abundance OR alteration OR restoration OR reconstitution OR recovery OR correction OR correct* OR disrupt*)) OR (OTU or OTUs OR "operational taxonomic unit*" OR dysbiosis OR dysbacteriosis OR "16S rRNA" OR "rRNA, 16S" OR "16S Ribosomal RNA" OR "RNA, 16S Ribosomal" OR "Ribosomal RNA, 16S" OR "16S rDNA" OR "rDNA, 16S" OR "16S Ribosomal DNA" OR "DNA, 16S Ribosomal" OR "Ribosomal DNA, 16S") AND random* |
