# Supplementary material for: Probiotic supplementation during antibiotic treatment is unjustified in maintaining the gut microbiome diversity: a systematic review and meta-analysis
Source: BMC Med. 2023 Jul 19;21:262. doi: 10.1186/s12916-023-02961-0 (PMC10355080; doi:10.1186/s12916-023-02961-0)
Supplement: Supplementary file 2 — Additional file 2: Table S1. PRISMA checklist 2020; Table S2. Definitions of gut microbiome diversity outcomes reported in the included studies; Table S3. The summarized results of taxonomic analysis of microbiome composition as measured immediately at the end of simultaneous antibiotic and probiotic treatment; Table S4. Outcomes of follow-up as reported in each study; Table S5. Risk of bias assessment for all outcomes - Assignment to intervention; Table S6. Risk of bias assessment for all outcomes - Adhering to intervention; Table S7. GRADE assessment for the meta-analyses of Shannon, Chao1 and Observed OTUs diversity indices. [file 12916_2023_2961_MOESM2_ESM.docx]

**Table S1** PRISMA checklist 2020

| **Section and Topic** | **Item #** | **Checklist item** | **Location where item is reported** |
| --- | --- | --- | --- |
| **TITLE** | | |  |
| Title | 1 | Identify the report as a systematic review. | Title page |
| **ABSTRACT** | | |  |
| Abstract | 2 | See the PRISMA 2020 for Abstracts checklist. | Page 3 |
| **INTRODUCTION** | | |  |
| Rationale | 3 | Describe the rationale for the review in the context of existing knowledge. | Page 4-6 |
| Objectives | 4 | Provide an explicit statement of the objective(s) or question(s) the review addresses. | Page 6 |
| **METHODS** | | |  |
| Eligibility criteria | 5 | Specify the inclusion and exclusion criteria for the review and how studies were grouped for the syntheses. | Page 6 |
| Information sources | 6 | Specify all databases, registers, websites, organisations, reference lists and other sources searched or consulted to identify studies. Specify the date when each source was last searched or consulted. | Page 7 |
| Search strategy | 7 | Present the full search strategies for all databases, registers, and websites, including any filters and limits used. | Page 7 and S2 Table |
| Selection process | 8 | Specify the methods used to decide whether a study met the inclusion criteria of the review, including how many reviewers screened each record and each report retrieved, whether they worked independently, and if applicable, details of automation tools used in the process. | Page 7 and Page 11 (Fig 1) |
| Data collection process | 9 | Specify the methods used to collect data from reports, including how many reviewers collected data from each report, whether they worked independently, any processes for obtaining or confirming data from study investigators, and if applicable, details of automation tools used in the process. | Page 7 |
| Data items | 10a | List and define all outcomes for which data were sought. Specify whether all results that were compatible with each outcome domain in each study were sought (e.g., for all measures, time points, analyses), and if not, the methods used to decide which results to collect. | Page 7 and S3 Table |
|  | 10b | List and define all other variables for which data were sought (e.g., participant and intervention characteristics, funding sources). Describe any assumptions made about any missing or unclear information. | Page 9-13 (Table 1) |
| Study risk of bias assessment | 11 | Specify the methods used to assess risk of bias in the included studies, including details of the tool(s) used, how many reviewers assessed each study and whether they worked independently, and if applicable, details of automation tools used in the process. | Page 9 and S6-7 Tables; S7-12 Fig |
| Effect measures | 12 | Specify for each outcome the effect measure(s) (e.g., risk ratio, mean difference) used in the synthesis or presentation of results. | Page 7-9 |
| Synthesis methods | 13a | Describe the processes used to decide which studies were eligible for each synthesis (e.g., tabulating the study intervention characteristics and comparing against the planned groups for each synthesis (item #5)). | Page 7-9 |
|  | 13b | Describe any methods required to prepare the data for presentation or synthesis, such as handling of missing summary statistics, or data conversions. | Page 7-9 |
|  | 13c | Describe any methods used to tabulate or visually display results of individual studies and syntheses. | Page 7-9 |
|  | 13d | Describe any methods used to synthesize results and provide a rationale for the choice(s). If meta-analysis was performed, describe the model(s), method(s) to identify the presence and extent of statistical heterogeneity, and software package(s) used. | Page 7-9 |
|  | 13e | Describe any methods used to explore possible causes of heterogeneity among study results (e.g., subgroup analysis, meta-regression). | Page 7-9 |
|  | 13f | Describe any sensitivity analyses conducted to assess robustness of the synthesized results. | Page 7-9 |
| Reporting bias assessment | 14 | Describe any methods used to assess risk of bias due to missing results in a synthesis (arising from reporting biases). | Page 9 and S6-7 Tables; S7-12 Fig |
| Certainty assessment | 15 | Describe any methods used to assess certainty (or confidence) in the body of evidence for an outcome. | Page 9 |
| **RESULTS** | | |  |
| Study selection | 16a | Describe the results of the search and selection process, from the number of records identified in the search to the number of studies included in the review, ideally using a flow diagram. | Page 9-11and Fig 1 |
|  | 16b | Cite studies that might appear to meet the inclusion criteria, but which were excluded, and explain why they were excluded. | Page 9-11 |
| Study characteristics | 17 | Cite each included study and present its characteristics. | Page 10-13 (Table 1) |
| Risk of bias in studies | 18 | Present assessments of risk of bias for each included study. | S6-7 Tables and S7-12 Fig |
| Results of individual studies | 19 | For all outcomes, present, for each study: (a) summary statistics for each group (where appropriate) and (b) an effect estimate and its precision (e.g. confidence/credible interval), ideally using structured tables or plots. | Page 12-14 Fig 2-4 and S1-6 Fig |
| Results of syntheses | 20a | For each synthesis, briefly summarise the characteristics and risk of bias among contributing studies. | Page 12-14 and S7-12 Fig |
|  | 20b | Present results of all statistical syntheses conducted. If meta-analysis was done, present for each the summary estimate and its precision (e.g., confidence/credible interval) and measures of statistical heterogeneity. If comparing groups, describe the direction of the effect. | Page 12-14 Fig 2-4 and S1-6 Fig |
|  | 20c | Present results of all investigations of possible causes of heterogeneity among study results. | Page 12-14 Fig 2-4 and S1-6 Fig |
|  | 20d | Present results of all sensitivity analyses conducted to assess the robustness of the synthesized results. | Page 12-14 Fig 2-4 and S1-6 Fig |
| Reporting biases | 21 | Present assessments of risk of bias due to missing results (arising from reporting biases) for each synthesis assessed. | Page 19 and S6-7 Tables and S7-12 Fig |
| Certainty of evidence | 22 | Present assessments of certainty (or confidence) in the body of evidence for each outcome assessed. | Page 19and S8 Table |
| **DISCUSSION** | | |  |
| Discussion | 23a | Provide a general interpretation of the results in the context of other evidence. | Page 19-22 |
|  | 23b | Discuss any limitations of the evidence included in the review. | Page 24-25 |
|  | 23c | Discuss any limitations of the review processes used. | Page 24-25 |
|  | 23d | Discuss implications of the results for practice, policy, and future research. | Page 22-24 |
| **OTHER INFORMATION** | | |  |
| Registration and protocol | 24a | Provide registration information for the review, including register name and registration number, or state that the review was not registered. | Page 6 |
|  | 24b | Indicate where the review protocol can be accessed, or state that a protocol was not prepared. | Page 6 |
|  | 24c | Describe and explain any amendments to information provided at registration or in the protocol. | Page 6 |
| Support | 25 | Describe sources of financial or non-financial support for the review, and the role of the funders or sponsors in the review. | Page 27-28 |
| Competing interests | 26 | Declare any competing interests of review authors. | Page 27-28 |
| Availability of data, code and other materials | 27 | Report which of the following are publicly available and where they can be found: template data collection forms; data extracted from included studies; data used for all analyses; analytic code; any other materials used in the review. | Page 27-28 |

**Table S2** Definitions of gut microbiome diversity outcomes reported in the included studies

| **α-diversity indices** | **Definition** | **Reference** |
| --- | --- | --- |
| Shannon diversity index | The Shannon diversity index shows how diverse the species in a given community are. It rises with the number of species and the evenness of their abundance. The higher the index is, the more diverse the species are in the habitat. If the index equals 0, only one species is present in the community. The index has no upper limit. According to the current data, there are no clearly defined reference values for the “ideal” Shannon diversity of the microbiome. For the definition of low diversity, the cut-off points in the literature range from 2.0 to 4.0. | (20-22) |
| Heip index | This index ranges from 0 to 1 and measures how equally the species richness contributes to the total abundance or biomass of the community. | (23) |
| Observed OTUs | An OTU table contains the number of sequences that are observed for each taxonomic unit (OTUs) in each sample. An OTU can be defined as a collection of 16S rRNA sequences that have a certain percentage of sequence divergence. Columns usually represent samples, and rows represent genera or species-specific taxonomic units (OTUs). | (24) |
| Chao1 index | Chao1 is a nonparametric method for estimating the number of species in a community. The Chao richness estimator is based on the concept that rare species infer the most information about the number of missing species. | (25) |
| PD whole tree / Faith Phylogenetic diversity | A quantitative measure of phylogenetic diversity, “PD”, has been defined as the minimum total length of all the phylogenetic branches required to span a given set of taxa on the phylogenetic tree. | (26) |
| Strong’s dominance index | Strong's dominance index measures the maximum departure between the observed proportions and a perfectly even community. | (27) |
| Pielou’s evenness | Pielou's evenness is an index that measures diversity along with species richness. While species richness is the number of different species in a given area, evenness is the count of individuals of each species in an area. A calculated value of Pielou's evenness ranges from 0 (no evenness) to 1 (complete evenness). | (28) |
| Sobs index | Sobs is the total number of species observed in a sample, or in a set of samples. | (29) |
| ACE (Abundance-based coverage estimator) of species richness index | The ACE is a nonparametric method for estimating the number of species using sample coverage, which is defined as the sum of the probabilities of the observed species. By the ACE method the groups can be categorized as abundant and rare groups according to the observed frequencies. | (30, 31) |
| **β-diversity indices** | **Definition** | **Reference** |
| Bray-Curtis (dis)similarity index | The Bray–Curtis dissimilarity is bounded between 0 and 1, where 0 means that the two sites have the same composition (i.e., they share all the species), and 1 means that the two sites do not share any species. At sites where BC is intermediate (e.g., BC = 0.5), this index differs from other commonly used indices. | (32) |
| Jaccard similarity coefficient | The Jaccard similarity index (sometimes called the Jaccard similarity coefficient) compares members of two sets to see which members are shared and which are distinct. It is a measure of similarity for the two sets of data, with a range from 0% to 100%. The higher the percentage, the more similar the two populations are. | (33) |
| Euclidean distance | When two samples are compared, Euclidean distance puts more weight on differences in species abundances than on difference in species presences. As a result, two samples not sharing any species could appear more similar (with lower Euclidean distance) than two samples which share species, but the species largely differ in their abundances | (34) |
| Canberra distance | Canberra distance calculates a sum of relative differences where the species-specific absolute values of difference are relativized by the sum of the numbers being compared, i.e., for each species-wise comparison the values are bounded [0, 1]. Because the absolute difference is relativized by the sum of the respective values, the value of a given arithmetic difference exhibits an extremely concave distribution declining from one and asymptotic to zero as values increase. | (35) |
| (un)weighted UniFrac distance | Both weighted (quantitative) and unweighted (qualitative) variants of UniFrac are widely used in microbial ecology, where the former accounts for the abundance of observed organisms, while the latter only considers their presence or absence. The distance is calculated between pairs of samples (each sample represents an organismal community). All taxa found in one or both samples are placed on a phylogenetic tree. A branch leading to taxa from both samples is marked as "shared" and branches leading to taxa which appear only in one sample are marked as "unshared". There is a weighted version of the UniFrac metric, which accounts for the relative abundance of each of the taxa within the communities. This is commonly used in metagenomic studies, where the number of metagenomic reads can be in the tens of thousands, and it is appropriate to 'bin' these reads into operational taxonomic units, or OTUs, which can then be dealt with as taxa within the UniFrac framework. | (36) |

Abbreviations: *OTU:* operational taxonomic unit; *PD*: phylogenetic diversity; *ACE:* abundance-based coverage estimator; *UniFrac*: unique fraction metric

**Table S3** The summarized results of taxonomic analysis of microbiome composition as measured immediately at the end of simultaneous antibiotic and probiotic treatment

| **Study** | **Changes in the intervention group** | **Changes in the control group** | **Comparison of the two groups immediately after cessation of the simultaneous antibiotic and probiotic treatment** |
| --- | --- | --- | --- |
| Studies with sequencing methods outcomes | | | |
| Cárdenas et al. (2020) | Family: *Clostridiales, Lachnospiracea*  Genus: *Bacteroides, Prevotella Lactobacillus*  Family: *Ruminococcaceae*  Genus: *Bacteroides* and other undefined genera OTUs | No difference observed | Family: A higher abundance of *Enterobacteriaceae* was found in the intervention group, compared to control group. |
| Chen et al. (2018) | Phylum: *Proteobacteria, Cyanobacteria, Actinobacteria*  Species: *Clostridium butyricum* (not statistically significant)  Phylum: *Firmicutes, Bacteroidetes, Verrucomicrobia, Tenericutes*  *Bacteroidetes:Firmicutes* ratio  Class: *Fusobacteria* | Phylum**:** *Proteobacteria, Cyanobacteria*  Family: *Enterobacteriaceae, Leuconostocaceae* Species: *Lactococcus raffinolactis, Lactobacillus sakei, Acinetobacter baumannii* NIPH60  Phylum: *Firmicutes, Bacteroidetes, Verrucomicrobia, Lentisphaerae*  *Bacteroidetes:Firmicutes* ratio  Family: *Lachnospiraceae, Ruminococcaceae, Rikkenellaceae, Christensenellaceae, Peptococcaceae, Clostridiales* Family XI., *Victivallaceae* | Species: *Clostridium butyricum* in probiotic group was significantly higher compared to control group. |
| De Wolfe et al. (2018) | Genus*: Bacteroides* | N.D. | N.D. |
| Kabbani et al. (2017) | Genus: *Escherichia, Parabacteroides, Enterobacter, Odoribacter, Stenotrophomonas* | Genus: *Escherichia, Parabacteroides, Enterobacter*  Genus: *Roseburia, Ruminococcus* | Genus*:* *Ralstonia* and *Proprionibacterium* levels were higher in probiotic group, whereas *Parabacteroides* level were higher in the antibiotic group. The increase in *Escherichia* prevalence was less (but not statistically significant) in the probiotic group. |
| Kakiuchi et al. (2020) | Genus: *Blautia* | *Genus*: *Collinsella* and *Bifidobacterium* | N.D. |
| MacPherson et al. (2018) | Family: *Bacteroidaceae Enterobacteriaceae, Porphyromonadaceae*  Family: *Coriobacteriaceae, Peptostreptococcaceae, Lachnospiraceae,* *Ruminococcaceae*, unidentifed *Clostridiales* | Family: *Bacteroidaceae Enterobacteriaceae, Porphyromonadaceae*  Family: *Coriobacteriaceae, Peptostreptococcaceae, Lachnospiraceae, Ruminococcaceae*, unidentifed *Clostridiales* | Family: In the family of *Porphyromonadaceae* (specifically the genus *Parabacteroides*) there was an increase in the probiotic group compared to the control. |
| Oh et al. (2015) | Phylum: *Proteobacteria*  Genus: *Escherichia*  Phylum: *Firmicutes* | Phylum: *Proteobacteria*  Genus: *Escherichia*  Phylum: *Firmicutes* | Phylum: The changed proportions of the phyla were larger in the antibiotics group than in the probiotics group.  Genus*:* Changed genera were larger in the antibiotics group. |
| Tang et al. (2020) | Phylum*:* *Proteobacteria*  Genus: *Shigella, Klebsiella, Streptococcus, Veillonella, Enterococcus, Citrobacter, Oscillospira*  Phylum: *Firmicutes, Baceriodetes*  *Bacteroidetes:Firmicutes* ratio  Genus: *Bacteroides, Faecalibacterium, Roseburia, Phascolarctobacterium, Blautia* | Phylum: *Proteobacteria*  Genus*: Shigella, Klebsiella, Streptococcus, Veillonella, Dialister, Anaerotruncus, and Megasphaera*  Phylum**:** *Firmicutes, Baceriodetes*  Genus: *Bacteroides, Faecalibacterium, Roseburia, Phascolarctobacterium, and Blautia* | N.D. |
| Zhong et al. (2021) | N.D. | Phylum: *Actinobacteria*  Genus: *Bifidobacterium, Erysipelatoclostridium, Blautia, Lactobacillus,* *Clostridium_sensu_stricto_1, Peptoclostridium, Propionibacterium, Staphylococcus, Parabacteroides, Bacillus, Bifdobacterium, Lactobacillus* | Phylum: Increased relative abundance of *Actinobacteria* and *Proteobacteria* were found in the probiotic group  Genus: Higher relative abundance of *Bifidobacterium* was found in the probiotic group. |
| Engelbrektson et al. (2009) | N.D. | Relative increase  Family: *Enterobacteraceae*  Genus: *Clostridium, Eubacterium, Bacteroides*  Relative decrease  Genus: *Bifidobacterium*  No statistical difference between the mean bacterial counts before antibiotic treatment compared to the days after antibiotic treatment was observed due to large subject-to-subject variability. | Family: Counts on MacConkey agar (*Enterobactereaceae*) were significantly higher in the probiotic group.  Genus: The counts on *Bifidobacterium* iodoacetate medium agar (*Bifidobacterium*) were significantly higher in the probiotic group). |
| Studies with bacterial count outcomes (standard microbiological methods) | | | |
| Madden et al. (2005) | Total anaerobes | Total facultative anaerobes  Family*: Enterobactereaceae* | Genus: Numbers of *Bacteroides* in probiotic group were significantly lower compared to the control group. |
| Plummer et al. (2005) | Yeast  Total bacterial count  Family: *Enterobacteriaceae*  Genus: *Bacteroides, Bifidobacteria* | Yeast, *Candida albicans*  Total bacterial count, Total facultative anaerobes  Family: *Enterobacteriaceae*  Genus: *Bacteroides, Bifidobacteria, Lactobacilli* | Yeast: The number of *Candida albicans* in the placebo group was significantly higher than in the probiotic group. |
| Forssten et al. (2014) | Family: *Enterobacteriaceae*  Total bacterial counts, *Clostridium* *cluster XIV* | Family: *Enterobacteriaceae*  Total bacterial counts, *Clostridium* *cluster XIV* | Species: The probiotic group had significantly higher levels of *Bifidabecterium lactis* and *Lactobacillus acidophilus* ATCC 700396 compared to the placebo group. |
| Wang et al. (2017) | Number of total aerobs  Genus*:* *Enterococcus* | Yeast  Number of total aerobs  Genus*:* *Enterococcus* | N.D. |
| Amarri et al. (2008) | No significcant difference | No significant difference | N.D. |


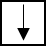
Legends:
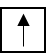
: increase in the number/proportion of given taxonomic group; : reduction in the number/proportion of given taxonomic group

Abbreviations: *N.D*.: no data

**Table S4** Outcomes of follow-up as reported in each study

| **Study** | **Duration of antibiotic treatment (days)** | **Duration of probiotic supplementation (days)** | **Whole study duration with follow-up (days)** | **Results of diversity indices at the end of follow-up period** | **Results of taxonomic composition at the end of follow-up period** |
| --- | --- | --- | --- | --- | --- |
| Cárdenas et al. (2020) | 14 | 14 | 45 | N.D. | The differences in relative abundance between the two groups were maintained. |
| Chen et al. (2018) | 14 | 14 | 56 | Sobs index: reduction maintained  Other diversity indices: showed no significant difference compared with baseline values. | Phylum*:* changes recovered to baseline in both groups  *Bacteroidetes:Firmicutes* ratio: normalized in both groups.  *Family*/*Genus:* Most significant changes  recovered except:   - *Rikkenellaceae, Christensenellaceae, Peptococcaceae, Clostridiales Family XI., Victivallaceae* in control group - *Ruminococcaceae, Lachnospiraceae and Eubacterium* in intervention group |
| De Wolfe et al. (2018) | 28 | 28 | 56 | N.D. | Family: *Verrucomicrobiaceae* was decreased in probiotic group compared to the control at week 8.  Genus: *Ruminococcus* level was elevated at week 8 in both groups. |
| Kabbani et al. (2017) | 7 | 14 | 21 | N.D. | Genus: Prevalence of *Escherichia* reduced to baseline in both groups.  *Ralstonia, Bradyrhizobium, Proprionibacterium, Cetobacterium, Anaerobiospirillim, Bulleidia* and *Stenotrophomonas* represented a higher prevalence in probiotic group compared to control. |
| MacPherson et al. (2018) | 7 | 14 | 28 | N.D. | Even one week after the cessation of the antimicrobial, microbiome compositions recovered to baseline in both groups which then maintained until the end of follow-up. |
| Tang et al. (2020) | 14 | 28 | 56 | Alpha diversity: The significant decrease in alpha diversity indices were maintained for up to week 4, but almost returned to the baseline at week 8. There were no significant differences between the two groups at any of the timepoints.  Unweighted UniFrac distance:  The significant differences maintained at 4, 6, and 8 weeks after treatment compared to the baseline within both groups (but not between). | Phylum*:* no significant difference was observed in phylum level at weeks 4, 6, and 8  *Bacteroidetes:Firmicutes* ratio: normalized from week 4 to week 8  Genus*: Enterococcus*, *Bacillus*, and  *Lactobacillales* were enriched in the probiotic  group at week 4, which disappeared to weeks 6 and 8, except *Lactobacillales*. |
| Zhong et al. (2021) | 7 | 7 | 42 | N.D. | Differences in relative abundances were still maintained after the 42 days of follow-up between the two groups.  Genus: *Bifidobacterium, Erysipelatoclostridium, Blautia, Lactobacillus, Clostridium_sensu_stricto_1, Peptoclostridium, and Propionibacterium*  increased with time, whereas  *Staphylococcus, Parabacteroides,* and *Bacillus* showed decreasing trends over time in the control group. |
| Engelbrektson et al. (2009) | 7 | 20 | 34 | Bray–Curtis similarity for individual subjects: By day 34 there was no longer a significant effect of antibiotics. While both groups exhibited a  similar trend toward increased similarity to the baseline  over days 21 through 34, the probiotic group exhibited a  larger increase in similarity to the baseline by the time  probiotic treatment ended at day 34 (at the significance level of 10%). However, for day 48 similarity to the baseline decreased significantly in both groups. | N.D. |
| Forssten et al. (2014) | 7 | 14 | 22 | N.D. | Family: *Enterobacteriaceae* levels fell to baseline and *Clostridium cluster XIV* levels remained reduced in both groups when compared to baseline but was significantly higher in the probiotic group. Genus changes normalized in both groups.  Species: *Bifidobacterium lactis* level remained below baseline, while *Lactobacillus acidophilus* increased to above baseline level in the placebo group. Both species remained higher in probiotic group at day 15, but no significant difference was observed between groups at day 22. |
| Madden et al. (2005) | 7 | 14 | 27 | N.D. | Total (facultative) anaerobes: The number of anaerobes was significantly higher in the control group compared to baseline. In case of probiotic group, numbers of these bacteria  restored between days 7 and 27.  Genus: *Bifidobacteria* counts were higher at day 12 than at baseline and at day 7), but there were no significant differences between the starting and re-growth populations (day 27). *Enterobacter* counts normalized at day 27. |
| Plummer et al. (2005) | 7 | 21 | 35 | N.D. | Total bacterial numbers: There were no significant differences between baseline and day 35 counts in any of the groups. |
| Wang et al. (2017) | 14 | 14 | 71 | N.D. | Yeast: Increased amount of Yeast maintained in the control group  All other changes disappeared. |


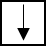
Legends:
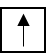
: increase in the number/proportion of given taxonomic group; : reduction in the number/proportion of given taxonomic group

Abbreviations: N.D.: no data

**Table S5** Risk of bias assessment for all outcomes - Assignment to intervention (the 'intention-to-treat' effect)

| Assignment to intervention (the 'intention-to-treat' effect) | | | |  |  |  |  |  |  |  |
| --- | --- | --- | --- | --- | --- | --- | --- | --- | --- | --- |
| **Reference** | **Intervention** | **Control** | **Outcome** | **Weight** | **Randomization process** | **Deviations from intented intervention** | **Missing outcome data** | **Measurement of the outcome** | **Selection of the reported results** | **Overall bias** |
| Cárdenas et al. | probiotics&antiobiotics | antibiotics | Shannon diversity index | 1 |  |  |  |  |  |  |
| Cárdenas et al. | probiotics&antiobiotics | antibiotics | Observed OTUs | 1 |  |  |  |  |  |  |
| Cárdenas et al. | probiotics&antibiotics | antibiotics | Faiths Phylogenetic Diversity | 1 |  |  |  |  |  |  |
| Cárdenas et al. | probiotics&antibiotics | antibiotics | Pielou’s evenness | 1 |  |  |  |  |  |  |
| Cárdenas et al. | probiotics&antibiotics | antibiotics | Bray-Curtis dissimilarity index | 1 |  |  |  |  |  |  |
| Cárdenas et al. | probiotics&antibiotics | antibiotics | Jaccard similarity coefficient | 1 |  |  |  |  |  |  |
| Cárdenas et al. | probiotics&antibiotics | antibiotics | Canberra distance | 1 |  |  |  |  |  |  |
| Cárdenas et al. | probiotics&antibiotics | antibiotics | weighted UniFrac distance | 1 |  |  |  |  |  |  |
| Cárdenas et al. | probiotics&antibiotics | antibiotics | unweighted UniFrac distance | 1 |  |  |  |  |  |  |
| Chen et al. | probiotics&antibiotics | antibiotics | Shannon diversity index | 1 |  |  |  |  |  |  |
| Chen et al. | probiotics&antibiotics | antibiotics | Heip index | 1 |  |  |  |  |  |  |
| Chen et al. | probiotics&antibiotics | antibiotics | Sobs index | 1 |  |  |  |  |  |  |
| DeWolfe et al. | probiotics&antibiotics | antibiotics | Shannon diversity index | 1 |  |  |  |  |  |  |
| DeWolfe et al. | probiotics&antibiotics | antibiotics | Bray-Curtis dissimilarity index | 1 |  |  |  |  |  |  |
| DeWolfe et al. | probiotics&antibiotics | antibiotics | Jaccard similarity coefficient | 1 |  |  |  |  |  |  |
| Engelbrektson et al. | probiotics&antibiotics | antibiotics | Bray-Curtis dissimilarity index | 1 |  |  |  |  |  |  |
| Engelbrektson et al. | probiotics&antibiotics | antibiotics | Eucledian distance | 1 |  |  |  |  |  |  |
| Kabbani et al. | probiotics&antibiotics | antibiotics | Observed OTUs | 1 |  |  |  |  |  |  |
| Kabbani et al. | probiotics&antibiotics | antibiotics | Chao1 index | 1 |  |  |  |  |  |  |
| Kabbani et al. | probiotics&antibiotics | antibiotics | ANOSIM | 1 |  |  |  |  |  |  |
| Kakiuchi et al. | probiotics&antibiotics | antibiotics | Shannon diversity index | 1 |  |  |  |  |  |  |
| Kakiuchi et al. | probiotics&antibiotics | antibiotics | Observed OTUs | 1 |  |  |  |  |  |  |
| Kakiuchi et al. | probiotics&antibiotics | antibiotics | Chao1 index | 1 |  |  |  |  |  |  |
| Kakiuchi et al. | probiotics&antibiotics | antibiotics | PD whole tree | 1 |  |  |  |  |  |  |
| Kakiuchi et al. | probiotics&antibiotics | antibotics | weighted UniFrac distance | 1 |  |  |  |  |  |  |
| Kakiuchi et al. | probiotics&antibiotics | antibiotics | unweighted UniFrac distance | 1 |  |  |  |  |  |  |
| MacPherson et al. | probiotics&antibiotics | antibiotics | Shannon diversity index | 1 |  |  |  |  |  |  |
| MacPherson et al. | probiotics&antibiotics | antibiotics | Observed OTUs | 1 |  |  |  |  |  |  |
| MacPherson et al. | probiotics&antibiotics | antibiotics | weighted UniFrac distance | 1 |  |  |  |  |  |  |
| Oh et al. | probiotics&antibiotics | antibiotics | Shannon diversity index | 1 |  |  |  |  |  |  |
| Oh et al. | probiotics&antibiotics | antibiotics | Observed OTUs | 1 |  |  |  |  |  |  |
| Oh et al. | probiotics&antibiotics | antibiotics | Chao1 index | 1 |  |  |  |  |  |  |
| Tang et al. | probiotics&antibiotics | antibiotics | Chao1 index | 1 |  |  |  |  |  |  |
| Tang et al. | probiotics&antibiotics | antibiotics | Faith Phylogenetic Diversity | 1 |  |  |  |  |  |  |
| Tang et al. | probiotics&antibiotics | antibiotics | Shannon diversity index | 1 |  |  |  |  |  |  |
| Tang et al. | probiotics&antibiotics | antibiotics | Observed OTUs | 1 |  |  |  |  |  |  |
| Tang et al. | probiotics&antibiotics | antibiotics | Singles index | 1 |  |  |  |  |  |  |
| Tang et al. | probiotics&antibiotics | antibiotics | Strong index | 1 |  |  |  |  |  |  |
| Tang et al. | probiotics&antibiotics | antibiotics | Unweighted UniFrac distance | 1 |  |  |  |  |  |  |
| Zhong et al. | probiotics&antibiotics | antibiotics | Shannon diversity index | 1 |  |  |  |  |  |  |
| Zhong et al. | probiotics&antibiotics | antibiotics | Observed OTUs | 1 |  |  |  |  |  |  |
| Zhong et al. | probiotics&antibiotics | antibiotics | Ace index | 1 |  |  |  |  |  |  |
| Amarri et al. | probiotics&antibiotics | antibiotics | Similarity coefficient | 1 |  |  |  |  |  |  |
| Forssten et al. | probiotics&antibiotics | antibiotics | log10 counts/g wet weight | 1 |  |  |  |  |  |  |
| Madden et al. | probiotics&antibiotics | antibiotics | log cfu/g dry weight of faeces | 1 |  |  |  |  |  |  |
| Plummer et al. | probiotics&antibiotics | antibiotics | log cfu/g dry weight of faeces | 1 |  |  |  |  |  |  |
| Wang et al. | probiotics&antibiotics | antibiotics | log10 counts/g wet weight | 1 |  |  |  |  |  |  |

**Table S6** Risk of bias assessment for all outcomes - Adhering to intervention (the 'per-protocol' effect)

| Adhering to intervention (the 'per-protocol' effect) | | | |  |  |  |  |  |  |  |
| --- | --- | --- | --- | --- | --- | --- | --- | --- | --- | --- |
| **Reference** | **Intervention** | **Control** | **Outcome** | **Weight** | **Randomization process** | **Deviations from intented intervention** | **Missing outcome data** | **Measurement of the outcome** | **Selection of the reported results** | **Overall bias** |
| Cárdenas et al. | probiotics&antibiotics | antibiotics | Shannon diversity index | 1 |  |  |  |  |  |  |
| Cárdenas et al. | probiotics&antibiotics | antibiotics | Observed OTUs | 1 |  |  |  |  |  |  |
| Cárdenas et al. | probiotics&antibiotics | antibiotics | Faiths Phylogenetic Diversity | 1 |  |  |  |  |  |  |
| Cárdenas et al. | probiotics&antibiotics | antibiotics | Pielou’s evenness | 1 |  |  |  |  |  |  |
| Cárdenas et al. | probiotics&antibiotics | antibiotics | Bray-Curtis dissimilarity index | 1 |  |  |  |  |  |  |
| Cárdenas et al. | probiotics&antibiotics | antibiotics | Jaccard similarity coefficient | 1 |  |  |  |  |  |  |
| Cárdenas et al. | probiotics&antibiotics | antibiotics | Canberra distance | 1 |  |  |  |  |  |  |
| Cárdenas et al. | probiotics&antibiotics | antibiotics | weighted UniFrac distance | 1 |  |  |  |  |  |  |
| Cárdenas et al. | probiotics&antibiotics | antibiotics | unweighted Unifrac distance | 1 |  |  |  |  |  |  |
| Chen et al. | probiotics&antibiotics | antibiotics | Shannon diversity index | 1 |  |  |  |  |  |  |
| Chen et al. | probiotics&antibiotics | antibiotics | Heip index | 1 |  |  |  |  |  |  |
| Chen et al. | probiotics&antibiotics | antibiotics | Sobs index | 1 |  |  |  |  |  |  |
| DeWolfe et al. | probiotics&antibiotics | antibiotics | Shannon diversity index | 1 |  |  |  |  |  |  |
| DeWolfe et al. | probiotics&antibiotics | antibiotics | Bray-Curtis dissimilarity index | 1 |  |  |  |  |  |  |
| DeWolfe et al. | probiotics&antibiotics | antibiotics | Jaccard similarity coefficient | 1 |  |  |  |  |  |  |
| Engelbrektson et al. | probiotics&antibiotics | antibiotics | Bray-Curtis dissimilarity index | 1 |  |  |  |  |  |  |
| Engelbrektson et al. | probiotics&antibiotics | antibiotics | Eucledian distance | 1 |  |  |  |  |  |  |
| Kabbani et al. | probiotics&antibiotics | antibiotics | Observed OTUs | 1 |  |  |  |  |  |  |
| Kabbani et al. | probiotics&antibiotics | antibiotics | Chao1 index | 1 |  |  |  |  |  |  |
| Kabbani et al. | probiotics&antibiotics | antibiotics | ANOSIM | 1 |  |  |  |  |  |  |
| Kakiuchi et al. | probiotics&antibiotics | antibiotics | Shannon diversity index | 1 |  |  |  |  |  |  |
| Kakiuchi et al. | probiotics&antibiotics | antibiotics | Observed OTUs | 1 |  |  |  |  |  |  |
| Kakiuchi et al. | probiotics&antibiotics | antibotics | Chao1 index | 1 |  |  |  |  |  |  |
| Kakiuchi et al. | probiotics&antibotics | antibiotics | PD whole tree | 1 |  |  |  |  |  |  |
| Kakiuchi et al. | probiotics&antibiotics | antibiotics | weighted UniFrac distance | 1 |  |  |  |  |  |  |
| Kakiuchi et al. | probiotics&antibiotics | antibiotics | unweighted UniFrac distance | 1 |  |  |  |  |  |  |
| MacPherson et al. | probiotics&antibiotics | antibiotics | Shannon diversity index | 1 |  |  |  |  |  |  |
| MacPherson et al. | probiotics&antibiotics | antibiotics | Observed OTUs | 1 |  |  |  |  |  |  |
| MacPherson et al. | probiotics&antibiotics | antibiotics | weighted UniFrac distance | 1 |  |  |  |  |  |  |
| Oh et al. | probiotics&antibiotics | antibiotics | Shannon diversity index | 1 |  |  |  |  |  |  |
| Oh et al. | probiotics&antibiotics | antibiotics | Observed OTUs | 1 |  |  |  |  |  |  |
| Oh et al. | probiotics&antibiotics | antibiotics | Chao1 index | 1 |  |  |  |  |  |  |
| Tang et al. | probiotics&antibiotics | antibiotics | Chao1 index | 1 |  |  |  |  |  |  |
| Tang et al. | probiotics&antibiotics | antibiotics | Shannon diversity index | 1 |  |  |  |  |  |  |
| Tang et al. | probiotics&antibiotics | antibiotics | Observed OTUs | 1 |  |  |  |  |  |  |
| Tang et al. | probiotics&antibiotics | antibiotics | Faith Phylogenetic Diversity | 1 |  |  |  |  |  |  |
| Tang et al. | probiotics&antibiotics | antibiotics | Singles index | 1 |  |  |  |  |  |  |
| Tang et al. | probiotics&antibiotics | antibiotics | Strong index | 1 |  |  |  |  |  |  |
| Tang et al. | probiotics&antibiotics | antibiotics | Unweighted UniFrac distance | 1 |  |  |  |  |  |  |
| Zhong et al. | probiotics&antibiotics | antibiotics | Shannon diversity index | 1 |  |  |  |  |  |  |
| Zhong et al. | probiotics&antibiotics | antibiotics | Observed OTUs | 1 |  |  |  |  |  |  |
| Zhong et al. | probiotics&antibiotics | antibiotics | Ace index | 1 |  |  |  |  |  |  |
| Amarri et al. | probiotics&antibiotics | antibiotics | Similarity coefficient | 1 |  |  |  |  |  |  |
| Forssten et al. | probiotics&antibiotics | antibiotics | log10 counts/g wet weight | 1 |  |  |  |  |  |  |
| Madden et al. | probiotics&antibiotics | antibiotics | log cfu/g dry weight of faeces | 1 |  |  |  |  |  |  |
| Plummer et al. | probiotics&antibiotics | antibiotics | log cfu/g dry weight of faeces | 1 |  |  |  |  |  |  |
| Wang et al. | probiotics&antibiotics | antibiotics | log10 counts/g wet weight | 1 |  |  |  |  |  |  |

**Table S7** GRADE assessment for the meta-analyses of Shannon, Chao1 and Observed OTUs diversity indices.

**Author(s):** Elias et al.

**Question:** Probiotics compared to no probiotics in antibiotic therapy

| **Certainty assessment** | | | | | | | **№ of patients** | | **Effect** | | **Certainty** | **Importance** |
| --- | --- | --- | --- | --- | --- | --- | --- | --- | --- | --- | --- | --- |
| **№ of studies** | **Study design** | **Risk of bias** | **Inconsistency** | **Indirectness** | **Imprecision** | **Other considerations** | **probiotics** | **no probiotics** | **Relative (95% CI)** | **Absolute (95% CI)** |  |  |
| **Shannon diversity index (follow-up: range 7 days to 28 days; assessed with: sequencing; Scale from: 0 to 10)** | | | | | | | | | | | | |
| 5 | randomised trials | serious | serious | not serious | serious | none | 171 | 164 | - | MD **0.23 Shannon diversity index higher** (0.06 lower to 0.51 higher) | ⨁⨁◯◯ Low | IMPORTANT |
| **Observed OTUs (follow-up: range 7 days to 14 days; assessed with: sequencing; Scale from: 0 to 1000)** | | | | | | | | | | | | |
| 3 | randomised trials | not serious | serious | not serious | serious | none | 121 | 115 | - | MD **17.15 Observed OTUs higher** (9.43 lower to 43.73 higher) | ⨁⨁◯◯ Low | IMPORTANT |
| **Chao1 index (follow-up: range 7 days to 14 days; assessed with: sequencing; Scale from: 0 to 1000)** | | | | | | | | | | | | |
| 3 | randomised trials | serious | serious | not serious | serious | none | 121 | 115 | - | MD **11.59 Chao1 index higher** (18.42 lower to 41.6 higher) | ⨁⨁◯◯ Low | IMPORTANT |

**CI:** confidence interval; **MD:** mean difference
