# Supplementary material for: Probiotic supplementation during antibiotic treatment is unjustified in maintaining the gut microbiome diversity: a systematic review and meta-analysis
Source: BMC Med. 2023 Jul 19;21:262. doi: 10.1186/s12916-023-02961-0 (PMC10355080; doi:10.1186/s12916-023-02961-0)
Supplement: Supplementary file 3 — Additional file 3: Fig. S1. Additional sensitivity analysis for the baseline values of Shannon diversity index; Fig. S2. Additional sensitivity analysis for the change between the “before-after” values of Shannon diversity index; Fig. S3. Additional sensitivity analysis for the baseline values of Chao1 index; Fig. S4. Additional sensitivity analysis for the change between the “before-after” values of Chao1 index; Fig. S5. Additional sensitivity analysis for the baseline values of Observed OTUs; Fig. S6. Additional sensitivity analysis for the change between the “before-after” values of Observed OTUs; Fig. S7. Risk of bias assessment for the main meta-analysis of Shannon diversity index - Assignment to intervention; Fig. S8. Risk of bias assessment for the main meta-analysis of Shannon diversity index - Adhering to intervention ; Fig. S9. Risk of bias assessment for the meta-analysis of Chao1 index - Assignment to intervention; Fig. S10. Risk of bias assessment for the meta-analysis of Chao1 index - Adhering to interventionFig. S11. Risk of bias assessment for the meta-analysis of Observed OTUs - Assignment to intervention; Fig. S12. Risk of bias assessment for the meta-analysis of Observed OTUs - Adhering to intervention. [file 12916_2023_2961_MOESM3_ESM.zip › Elias-et-al._Additional File 3R2.docx]

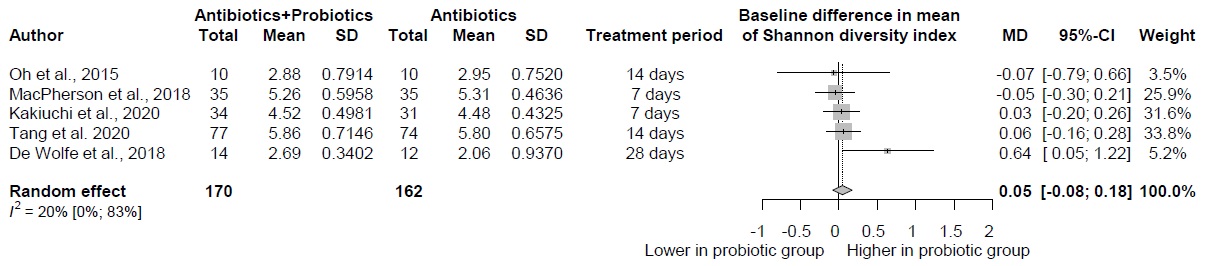


**Fig. S1** Additional sensitivity analysis for the baseline values of Shannon diversity index. Abbreviations: SD: standard deviation CI: confidence interval


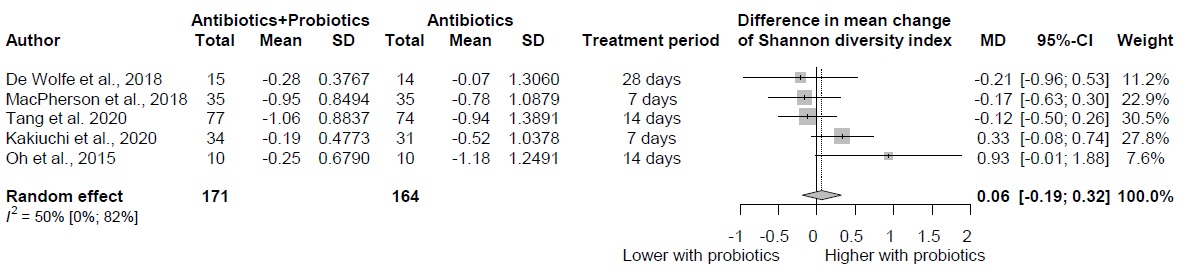


**Fig. S2** Additional sensitivity analysis for the change between the “before-after” values of Shannon diversity index. Abbreviations: SD: standard deviation CI: confidence interval


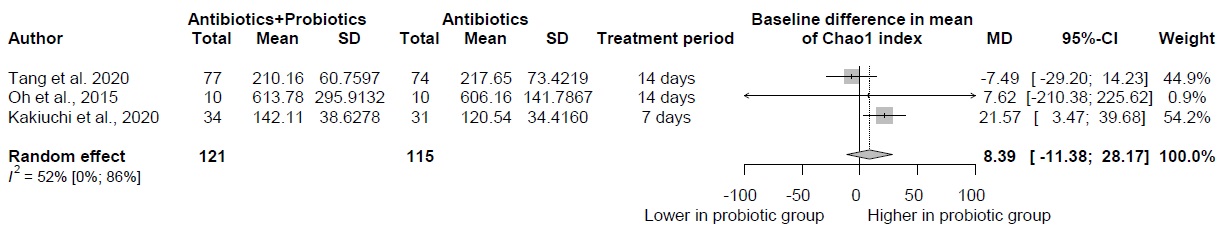


**Fig. S3** Additional sensitivity analysis for the baseline values of Chao1 index. Abbreviations: SD: standard deviation CI: confidence interval


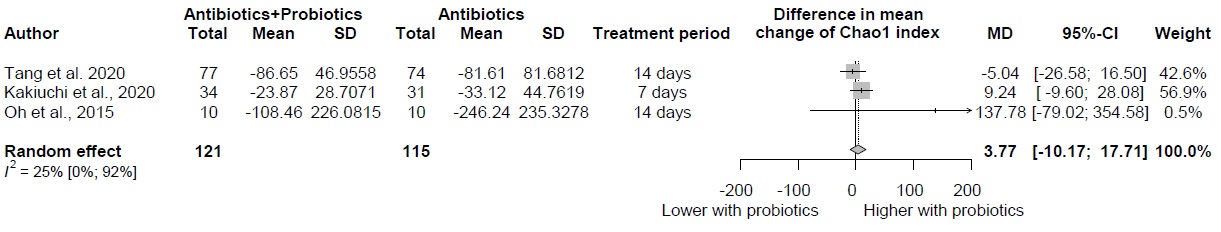


**Fig. S4** Additional sensitivity analysis for the change between the “before-after” values of Chao1 index. Abbreviations: SD: standard deviation CI: confidence interval


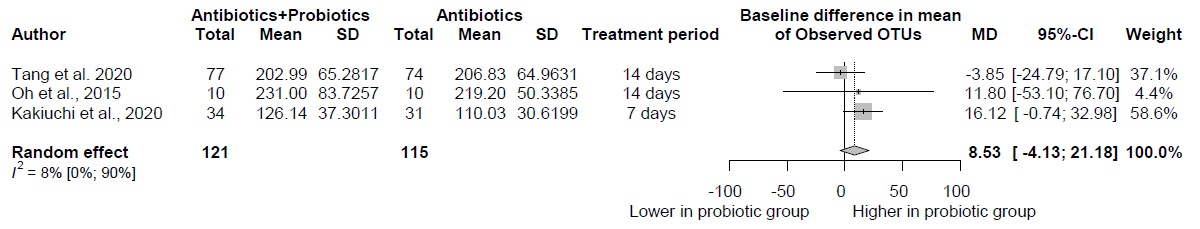


**Fig. S5** Additional sensitivity analysis for the baseline values of Observed OTUs. Abbreviations: SD: standard deviation CI: confidence interval OTU: Operational Taxonomic Unit


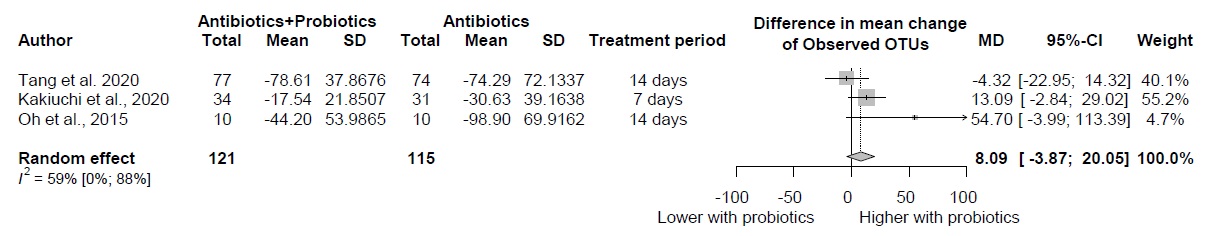


**Fig. S6** Additional sensitivity analysis for the change between the “before-after” values of Observed OTUs. Abbreviations: SD: standard deviation CI: confidence interval OTU: Operational Taxonomic Unit

**Fig. S7** Risk of bias assessment for the main meta-analysis of Shannon diversity index - Assignment to intervention (the “intention-to-treat” effect) (n=5)

**Fig. S8** Risk of bias assessment for the main meta-analysis of Shannon diversity index - Adhering to intervention (the “per-protocol” effect)

**Fig. S9** Risk of bias assessment for the meta-analysis of Chao1 index - Assignment to intervention (the “intention-to-treat” effect)

**Fig. 10** Risk of bias assessment for the meta-analysis of Chao1 index - Adhering to intervention (the “per-protocol” effect)

**Fig. S11** Risk of bias assessment for the meta-analysis of Observed OTUs - Assignment to intervention (the “intention-to-treat” effect)

**Fig. S12** Risk of bias assessment for the meta-analysis of Observed OTUs - Adhering to intervention (the “per-protocol”' effect)
