# Supplementary figures and images for: Probiotic supplementation during antibiotic treatment is unjustified in maintaining the gut microbiome diversity: a systematic review and meta-analysis
Source: BMC Med. 2023 Jul 19;21:262. doi: 10.1186/s12916-023-02961-0 (PMC10355080; doi:10.1186/s12916-023-02961-0)

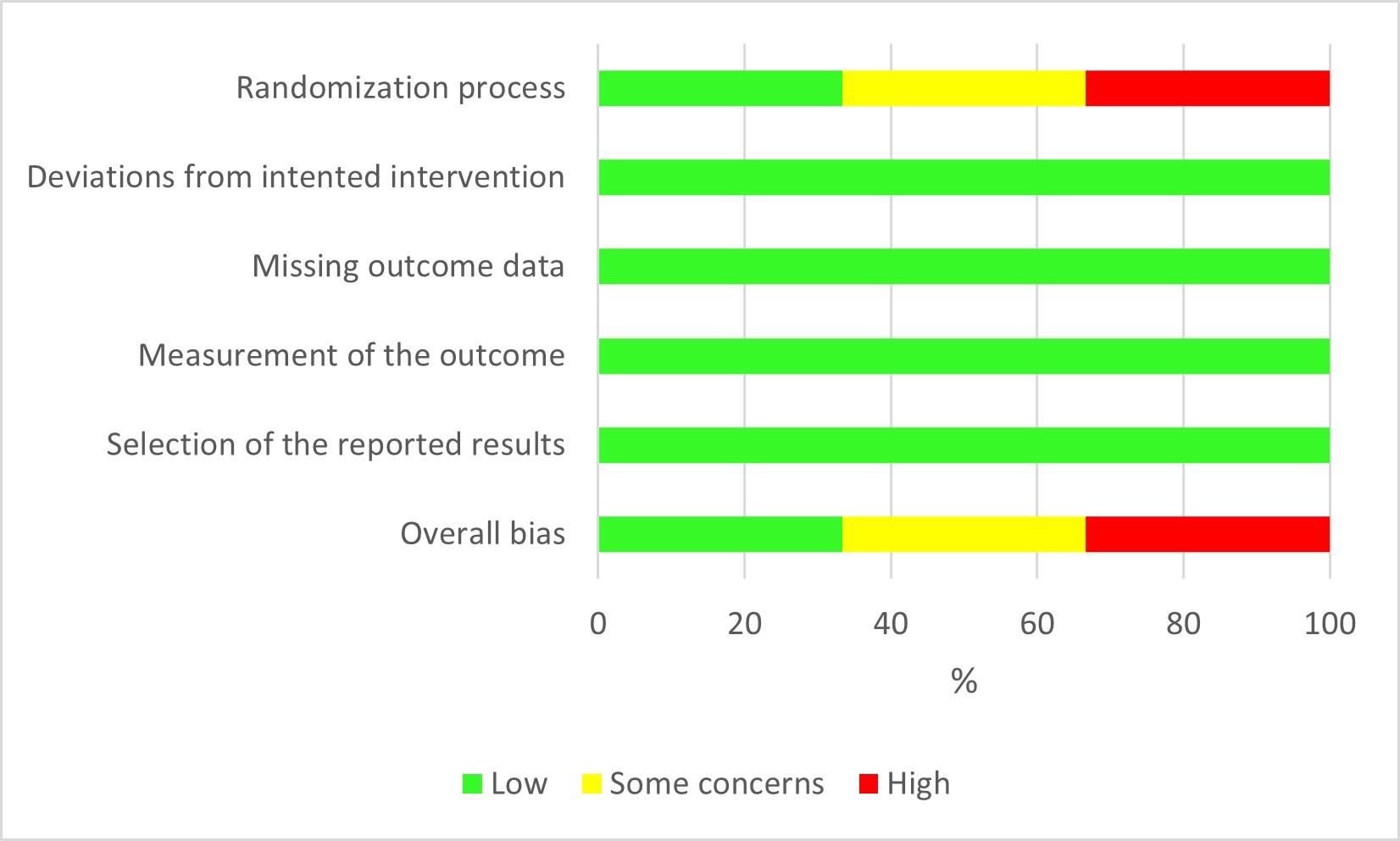

Supplement: Supplementary file 3 — Additional file 3: Fig. S1. Additional sensitivity analysis for the baseline values of Shannon diversity index; Fig. S2. Additional sensitivity analysis for the change between the “before-after” values of Shannon diversity index; Fig. S3. Additional sensitivity analysis for the baseline values of Chao1 index; Fig. S4. Additional sensitivity analysis for the change between the “before-after” values of Chao1 index; Fig. S5. Additional sensitivity analysis for the baseline values of Observed OTUs; Fig. S6. Additional sensitivity analysis for the change between the “before-after” values of Observed OTUs; Fig. S7. Risk of bias assessment for the main meta-analysis of Shannon diversity index - Assignment to intervention; Fig. S8. Risk of bias assessment for the main meta-analysis of Shannon diversity index - Adhering to intervention ; Fig. S9. Risk of bias assessment for the meta-analysis of Chao1 index - Assignment to intervention; Fig. S10. Risk of bias assessment for the meta-analysis of Chao1 index - Adhering to interventionFig. S11. Risk of bias assessment for the meta-analysis of Observed OTUs - Assignment to intervention; Fig. S12. Risk of bias assessment for the meta-analysis of Observed OTUs - Adhering to intervention. [file 12916_2023_2961_MOESM3_ESM.zip › Fig. S10R2.png]

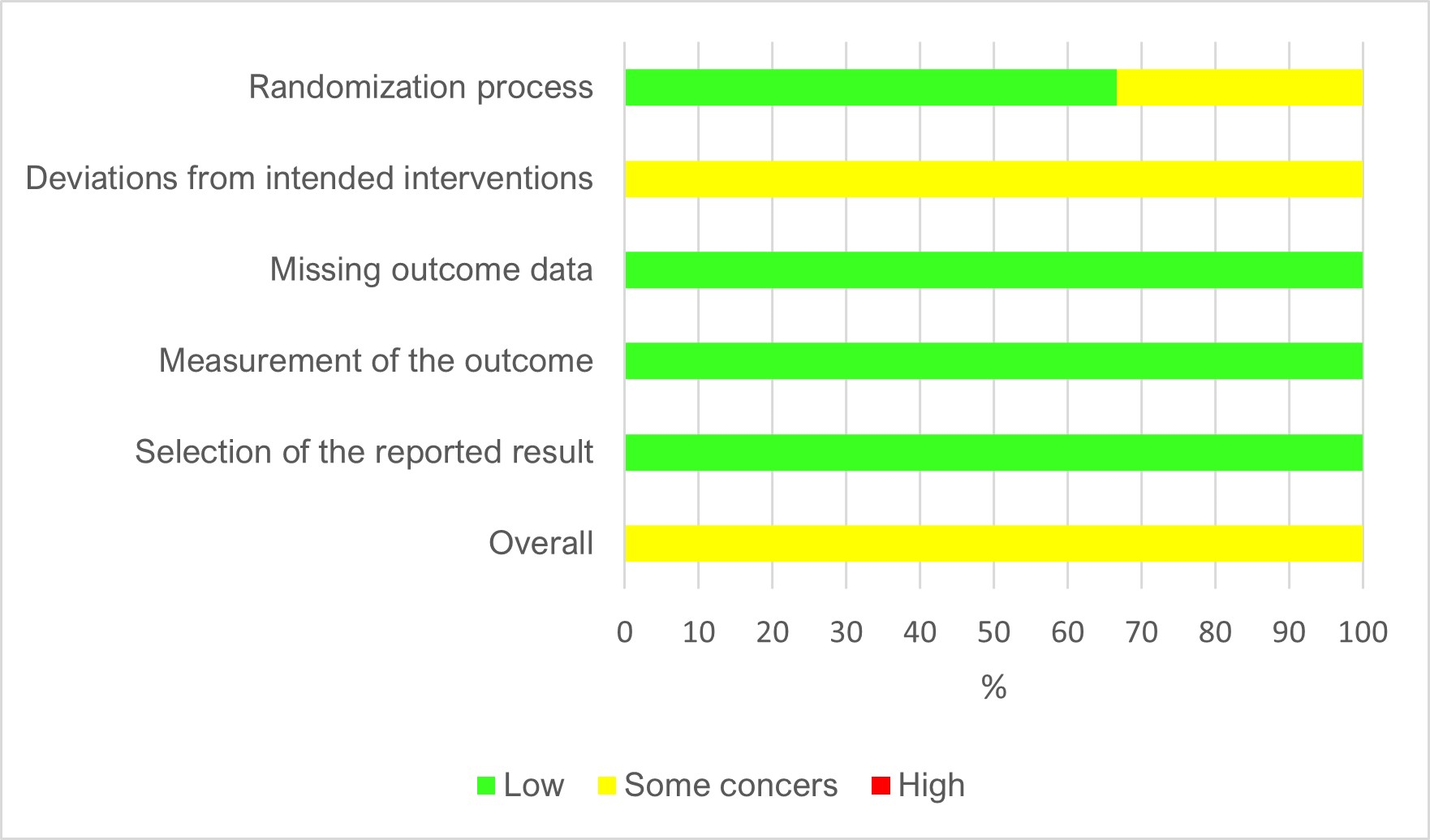

Supplement: Supplementary file 3 — Additional file 3: Fig. S1. Additional sensitivity analysis for the baseline values of Shannon diversity index; Fig. S2. Additional sensitivity analysis for the change between the “before-after” values of Shannon diversity index; Fig. S3. Additional sensitivity analysis for the baseline values of Chao1 index; Fig. S4. Additional sensitivity analysis for the change between the “before-after” values of Chao1 index; Fig. S5. Additional sensitivity analysis for the baseline values of Observed OTUs; Fig. S6. Additional sensitivity analysis for the change between the “before-after” values of Observed OTUs; Fig. S7. Risk of bias assessment for the main meta-analysis of Shannon diversity index - Assignment to intervention; Fig. S8. Risk of bias assessment for the main meta-analysis of Shannon diversity index - Adhering to intervention ; Fig. S9. Risk of bias assessment for the meta-analysis of Chao1 index - Assignment to intervention; Fig. S10. Risk of bias assessment for the meta-analysis of Chao1 index - Adhering to interventionFig. S11. Risk of bias assessment for the meta-analysis of Observed OTUs - Assignment to intervention; Fig. S12. Risk of bias assessment for the meta-analysis of Observed OTUs - Adhering to intervention. [file 12916_2023_2961_MOESM3_ESM.zip › Fig. S11R2.png]

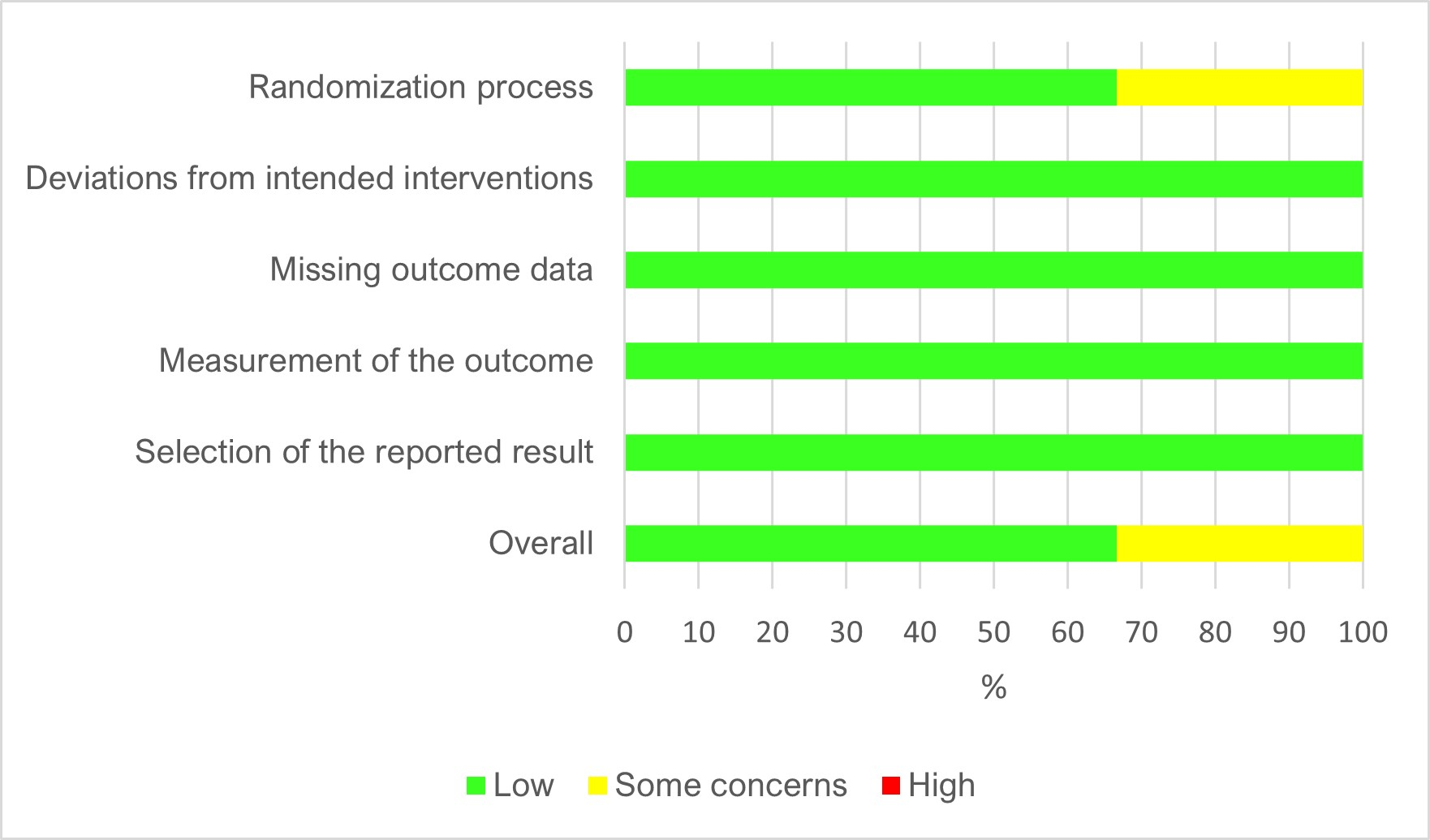

Supplement: Supplementary file 3 — Additional file 3: Fig. S1. Additional sensitivity analysis for the baseline values of Shannon diversity index; Fig. S2. Additional sensitivity analysis for the change between the “before-after” values of Shannon diversity index; Fig. S3. Additional sensitivity analysis for the baseline values of Chao1 index; Fig. S4. Additional sensitivity analysis for the change between the “before-after” values of Chao1 index; Fig. S5. Additional sensitivity analysis for the baseline values of Observed OTUs; Fig. S6. Additional sensitivity analysis for the change between the “before-after” values of Observed OTUs; Fig. S7. Risk of bias assessment for the main meta-analysis of Shannon diversity index - Assignment to intervention; Fig. S8. Risk of bias assessment for the main meta-analysis of Shannon diversity index - Adhering to intervention ; Fig. S9. Risk of bias assessment for the meta-analysis of Chao1 index - Assignment to intervention; Fig. S10. Risk of bias assessment for the meta-analysis of Chao1 index - Adhering to interventionFig. S11. Risk of bias assessment for the meta-analysis of Observed OTUs - Assignment to intervention; Fig. S12. Risk of bias assessment for the meta-analysis of Observed OTUs - Adhering to intervention. [file 12916_2023_2961_MOESM3_ESM.zip › Fig. S12R2.png]

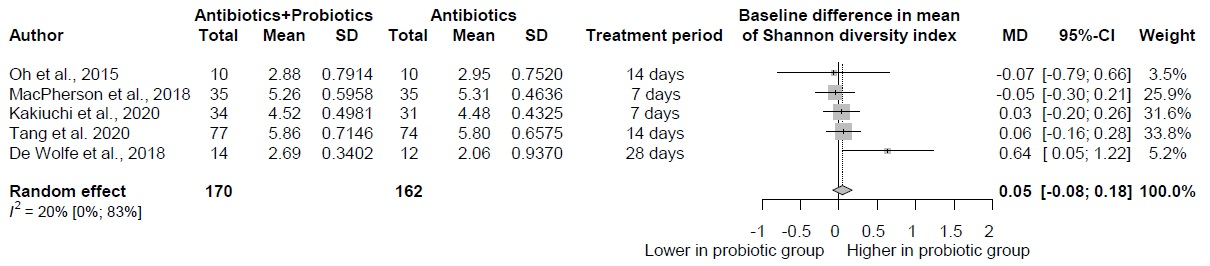

Supplement: Supplementary file 3 — Additional file 3: Fig. S1. Additional sensitivity analysis for the baseline values of Shannon diversity index; Fig. S2. Additional sensitivity analysis for the change between the “before-after” values of Shannon diversity index; Fig. S3. Additional sensitivity analysis for the baseline values of Chao1 index; Fig. S4. Additional sensitivity analysis for the change between the “before-after” values of Chao1 index; Fig. S5. Additional sensitivity analysis for the baseline values of Observed OTUs; Fig. S6. Additional sensitivity analysis for the change between the “before-after” values of Observed OTUs; Fig. S7. Risk of bias assessment for the main meta-analysis of Shannon diversity index - Assignment to intervention; Fig. S8. Risk of bias assessment for the main meta-analysis of Shannon diversity index - Adhering to intervention ; Fig. S9. Risk of bias assessment for the meta-analysis of Chao1 index - Assignment to intervention; Fig. S10. Risk of bias assessment for the meta-analysis of Chao1 index - Adhering to interventionFig. S11. Risk of bias assessment for the meta-analysis of Observed OTUs - Assignment to intervention; Fig. S12. Risk of bias assessment for the meta-analysis of Observed OTUs - Adhering to intervention. [file 12916_2023_2961_MOESM3_ESM.zip › Fig. S1R2.png]

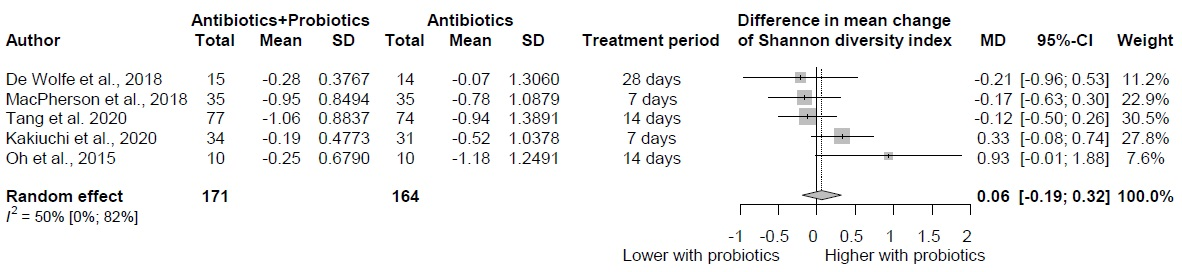

Supplement: Supplementary file 3 — Additional file 3: Fig. S1. Additional sensitivity analysis for the baseline values of Shannon diversity index; Fig. S2. Additional sensitivity analysis for the change between the “before-after” values of Shannon diversity index; Fig. S3. Additional sensitivity analysis for the baseline values of Chao1 index; Fig. S4. Additional sensitivity analysis for the change between the “before-after” values of Chao1 index; Fig. S5. Additional sensitivity analysis for the baseline values of Observed OTUs; Fig. S6. Additional sensitivity analysis for the change between the “before-after” values of Observed OTUs; Fig. S7. Risk of bias assessment for the main meta-analysis of Shannon diversity index - Assignment to intervention; Fig. S8. Risk of bias assessment for the main meta-analysis of Shannon diversity index - Adhering to intervention ; Fig. S9. Risk of bias assessment for the meta-analysis of Chao1 index - Assignment to intervention; Fig. S10. Risk of bias assessment for the meta-analysis of Chao1 index - Adhering to interventionFig. S11. Risk of bias assessment for the meta-analysis of Observed OTUs - Assignment to intervention; Fig. S12. Risk of bias assessment for the meta-analysis of Observed OTUs - Adhering to intervention. [file 12916_2023_2961_MOESM3_ESM.zip › Fig. S2R2.png]

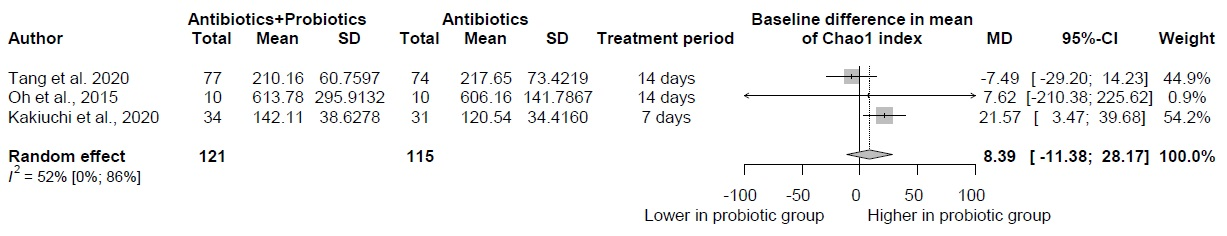

Supplement: Supplementary file 3 — Additional file 3: Fig. S1. Additional sensitivity analysis for the baseline values of Shannon diversity index; Fig. S2. Additional sensitivity analysis for the change between the “before-after” values of Shannon diversity index; Fig. S3. Additional sensitivity analysis for the baseline values of Chao1 index; Fig. S4. Additional sensitivity analysis for the change between the “before-after” values of Chao1 index; Fig. S5. Additional sensitivity analysis for the baseline values of Observed OTUs; Fig. S6. Additional sensitivity analysis for the change between the “before-after” values of Observed OTUs; Fig. S7. Risk of bias assessment for the main meta-analysis of Shannon diversity index - Assignment to intervention; Fig. S8. Risk of bias assessment for the main meta-analysis of Shannon diversity index - Adhering to intervention ; Fig. S9. Risk of bias assessment for the meta-analysis of Chao1 index - Assignment to intervention; Fig. S10. Risk of bias assessment for the meta-analysis of Chao1 index - Adhering to interventionFig. S11. Risk of bias assessment for the meta-analysis of Observed OTUs - Assignment to intervention; Fig. S12. Risk of bias assessment for the meta-analysis of Observed OTUs - Adhering to intervention. [file 12916_2023_2961_MOESM3_ESM.zip › Fig. S3R2.png]

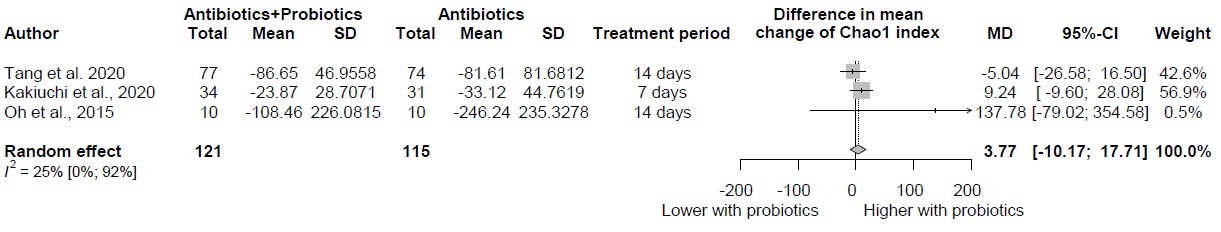

Supplement: Supplementary file 3 — Additional file 3: Fig. S1. Additional sensitivity analysis for the baseline values of Shannon diversity index; Fig. S2. Additional sensitivity analysis for the change between the “before-after” values of Shannon diversity index; Fig. S3. Additional sensitivity analysis for the baseline values of Chao1 index; Fig. S4. Additional sensitivity analysis for the change between the “before-after” values of Chao1 index; Fig. S5. Additional sensitivity analysis for the baseline values of Observed OTUs; Fig. S6. Additional sensitivity analysis for the change between the “before-after” values of Observed OTUs; Fig. S7. Risk of bias assessment for the main meta-analysis of Shannon diversity index - Assignment to intervention; Fig. S8. Risk of bias assessment for the main meta-analysis of Shannon diversity index - Adhering to intervention ; Fig. S9. Risk of bias assessment for the meta-analysis of Chao1 index - Assignment to intervention; Fig. S10. Risk of bias assessment for the meta-analysis of Chao1 index - Adhering to interventionFig. S11. Risk of bias assessment for the meta-analysis of Observed OTUs - Assignment to intervention; Fig. S12. Risk of bias assessment for the meta-analysis of Observed OTUs - Adhering to intervention. [file 12916_2023_2961_MOESM3_ESM.zip › Fig. S4R2.png]

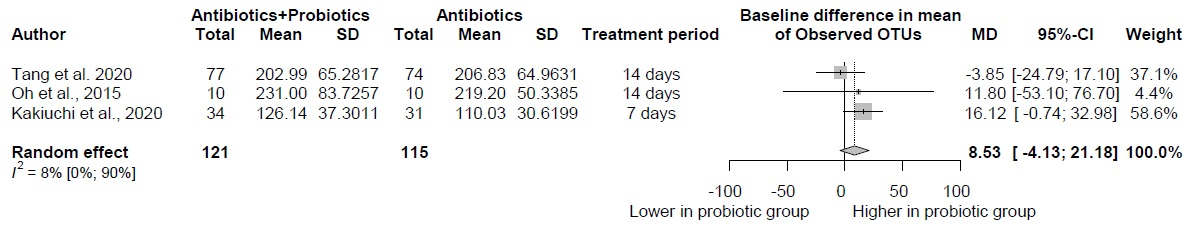

Supplement: Supplementary file 3 — Additional file 3: Fig. S1. Additional sensitivity analysis for the baseline values of Shannon diversity index; Fig. S2. Additional sensitivity analysis for the change between the “before-after” values of Shannon diversity index; Fig. S3. Additional sensitivity analysis for the baseline values of Chao1 index; Fig. S4. Additional sensitivity analysis for the change between the “before-after” values of Chao1 index; Fig. S5. Additional sensitivity analysis for the baseline values of Observed OTUs; Fig. S6. Additional sensitivity analysis for the change between the “before-after” values of Observed OTUs; Fig. S7. Risk of bias assessment for the main meta-analysis of Shannon diversity index - Assignment to intervention; Fig. S8. Risk of bias assessment for the main meta-analysis of Shannon diversity index - Adhering to intervention ; Fig. S9. Risk of bias assessment for the meta-analysis of Chao1 index - Assignment to intervention; Fig. S10. Risk of bias assessment for the meta-analysis of Chao1 index - Adhering to interventionFig. S11. Risk of bias assessment for the meta-analysis of Observed OTUs - Assignment to intervention; Fig. S12. Risk of bias assessment for the meta-analysis of Observed OTUs - Adhering to intervention. [file 12916_2023_2961_MOESM3_ESM.zip › Fig. S5R2.png]

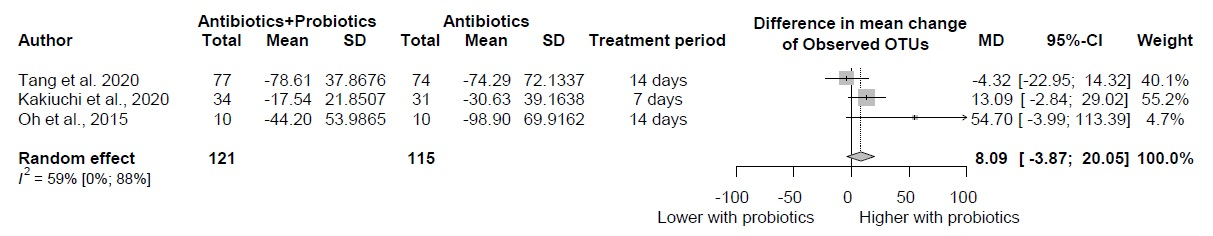

Supplement: Supplementary file 3 — Additional file 3: Fig. S1. Additional sensitivity analysis for the baseline values of Shannon diversity index; Fig. S2. Additional sensitivity analysis for the change between the “before-after” values of Shannon diversity index; Fig. S3. Additional sensitivity analysis for the baseline values of Chao1 index; Fig. S4. Additional sensitivity analysis for the change between the “before-after” values of Chao1 index; Fig. S5. Additional sensitivity analysis for the baseline values of Observed OTUs; Fig. S6. Additional sensitivity analysis for the change between the “before-after” values of Observed OTUs; Fig. S7. Risk of bias assessment for the main meta-analysis of Shannon diversity index - Assignment to intervention; Fig. S8. Risk of bias assessment for the main meta-analysis of Shannon diversity index - Adhering to intervention ; Fig. S9. Risk of bias assessment for the meta-analysis of Chao1 index - Assignment to intervention; Fig. S10. Risk of bias assessment for the meta-analysis of Chao1 index - Adhering to interventionFig. S11. Risk of bias assessment for the meta-analysis of Observed OTUs - Assignment to intervention; Fig. S12. Risk of bias assessment for the meta-analysis of Observed OTUs - Adhering to intervention. [file 12916_2023_2961_MOESM3_ESM.zip › Fig. S6R2.png]

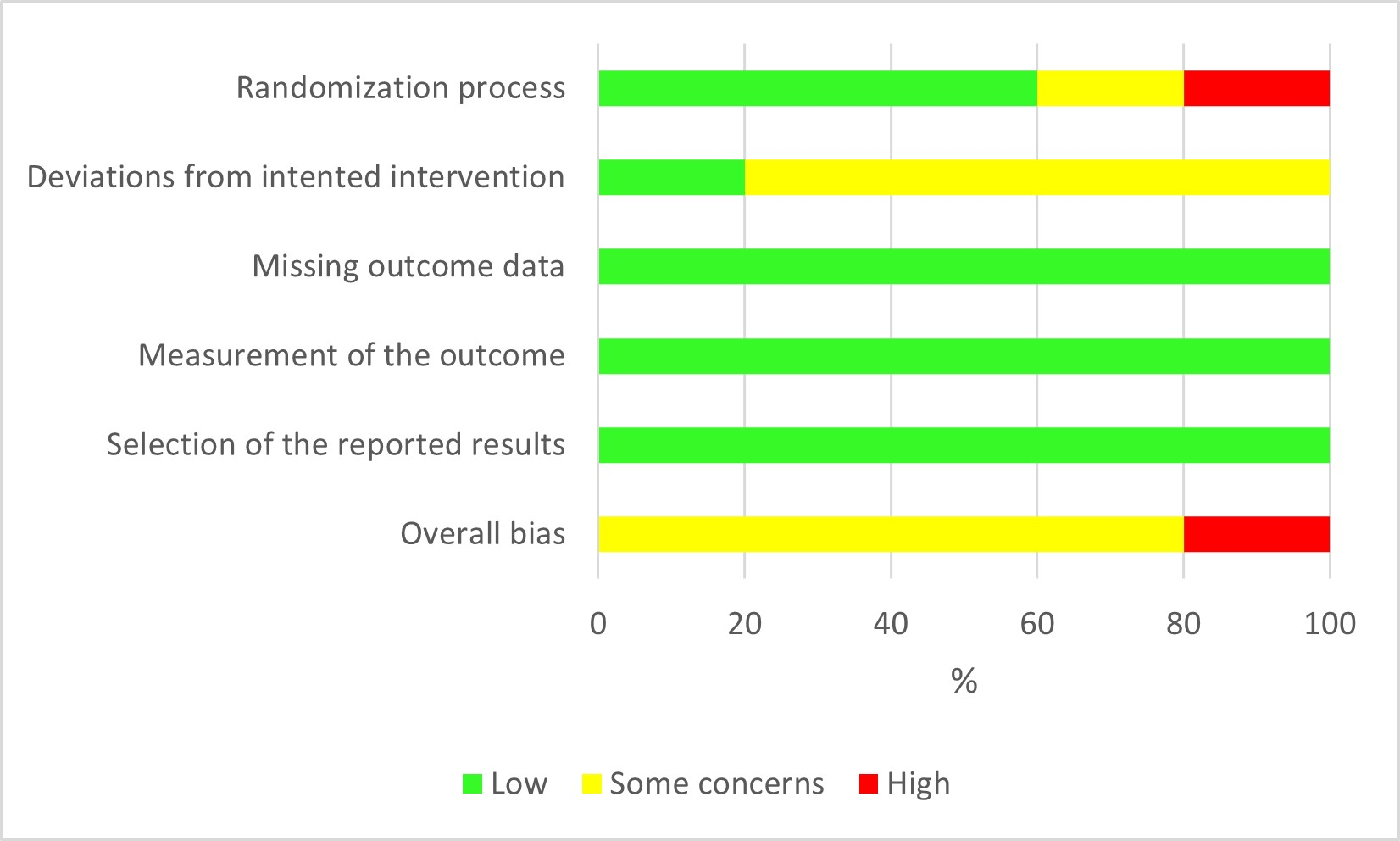

Supplement: Supplementary file 3 — Additional file 3: Fig. S1. Additional sensitivity analysis for the baseline values of Shannon diversity index; Fig. S2. Additional sensitivity analysis for the change between the “before-after” values of Shannon diversity index; Fig. S3. Additional sensitivity analysis for the baseline values of Chao1 index; Fig. S4. Additional sensitivity analysis for the change between the “before-after” values of Chao1 index; Fig. S5. Additional sensitivity analysis for the baseline values of Observed OTUs; Fig. S6. Additional sensitivity analysis for the change between the “before-after” values of Observed OTUs; Fig. S7. Risk of bias assessment for the main meta-analysis of Shannon diversity index - Assignment to intervention; Fig. S8. Risk of bias assessment for the main meta-analysis of Shannon diversity index - Adhering to intervention ; Fig. S9. Risk of bias assessment for the meta-analysis of Chao1 index - Assignment to intervention; Fig. S10. Risk of bias assessment for the meta-analysis of Chao1 index - Adhering to interventionFig. S11. Risk of bias assessment for the meta-analysis of Observed OTUs - Assignment to intervention; Fig. S12. Risk of bias assessment for the meta-analysis of Observed OTUs - Adhering to intervention. [file 12916_2023_2961_MOESM3_ESM.zip › Fig. S7R2.png]

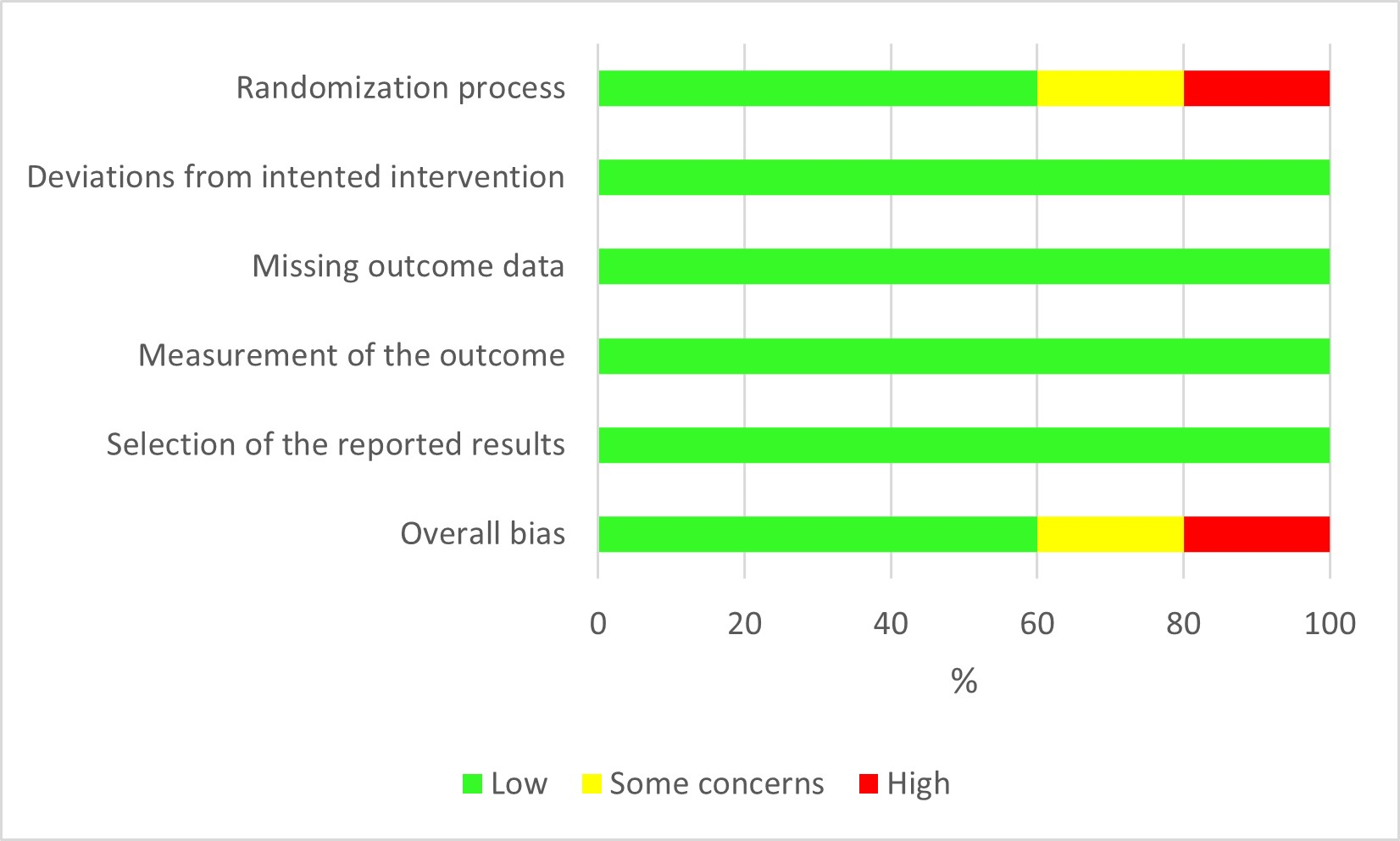

Supplement: Supplementary file 3 — Additional file 3: Fig. S1. Additional sensitivity analysis for the baseline values of Shannon diversity index; Fig. S2. Additional sensitivity analysis for the change between the “before-after” values of Shannon diversity index; Fig. S3. Additional sensitivity analysis for the baseline values of Chao1 index; Fig. S4. Additional sensitivity analysis for the change between the “before-after” values of Chao1 index; Fig. S5. Additional sensitivity analysis for the baseline values of Observed OTUs; Fig. S6. Additional sensitivity analysis for the change between the “before-after” values of Observed OTUs; Fig. S7. Risk of bias assessment for the main meta-analysis of Shannon diversity index - Assignment to intervention; Fig. S8. Risk of bias assessment for the main meta-analysis of Shannon diversity index - Adhering to intervention ; Fig. S9. Risk of bias assessment for the meta-analysis of Chao1 index - Assignment to intervention; Fig. S10. Risk of bias assessment for the meta-analysis of Chao1 index - Adhering to interventionFig. S11. Risk of bias assessment for the meta-analysis of Observed OTUs - Assignment to intervention; Fig. S12. Risk of bias assessment for the meta-analysis of Observed OTUs - Adhering to intervention. [file 12916_2023_2961_MOESM3_ESM.zip › Fig. S8R2.png]

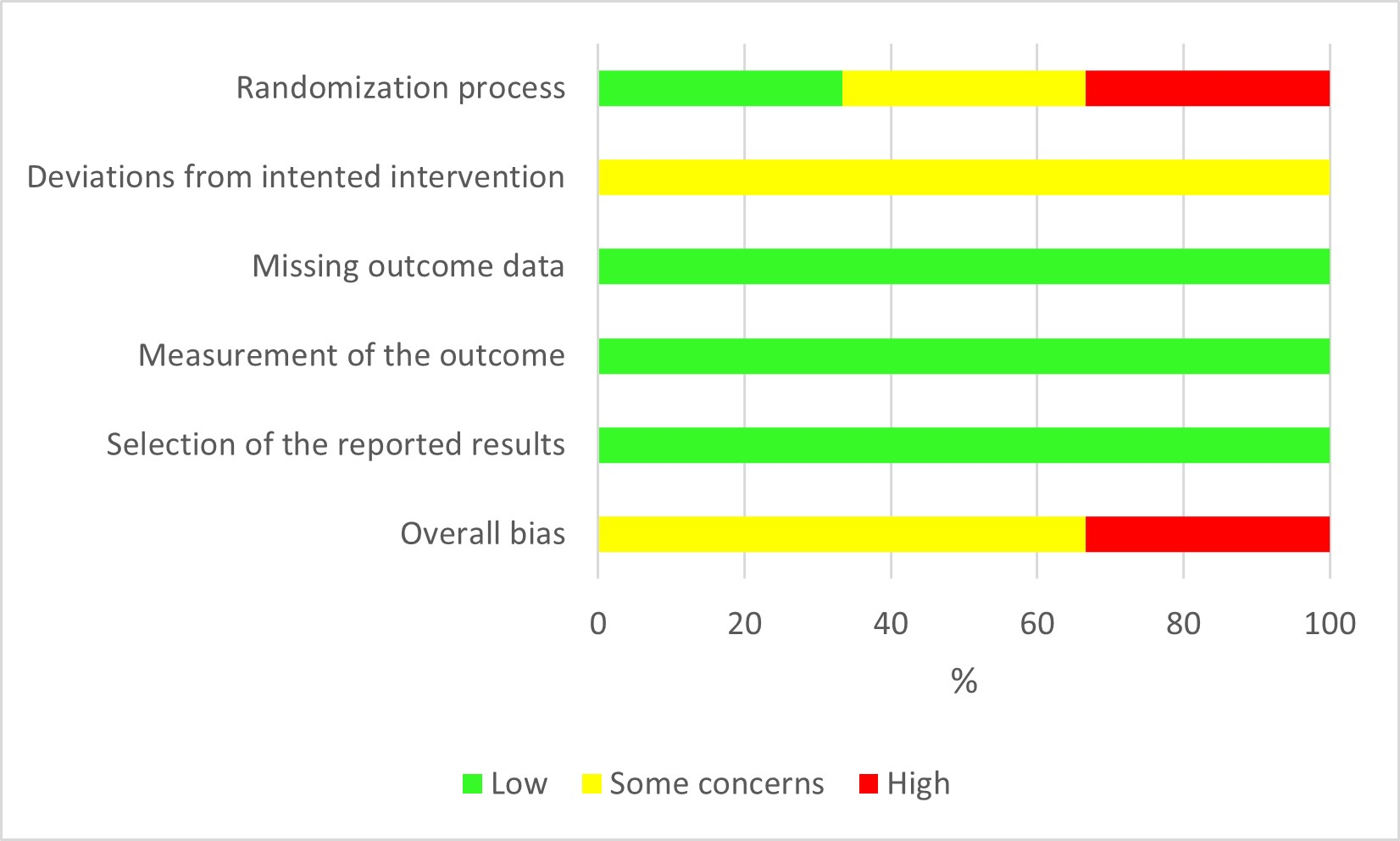

Supplement: Supplementary file 3 — Additional file 3: Fig. S1. Additional sensitivity analysis for the baseline values of Shannon diversity index; Fig. S2. Additional sensitivity analysis for the change between the “before-after” values of Shannon diversity index; Fig. S3. Additional sensitivity analysis for the baseline values of Chao1 index; Fig. S4. Additional sensitivity analysis for the change between the “before-after” values of Chao1 index; Fig. S5. Additional sensitivity analysis for the baseline values of Observed OTUs; Fig. S6. Additional sensitivity analysis for the change between the “before-after” values of Observed OTUs; Fig. S7. Risk of bias assessment for the main meta-analysis of Shannon diversity index - Assignment to intervention; Fig. S8. Risk of bias assessment for the main meta-analysis of Shannon diversity index - Adhering to intervention ; Fig. S9. Risk of bias assessment for the meta-analysis of Chao1 index - Assignment to intervention; Fig. S10. Risk of bias assessment for the meta-analysis of Chao1 index - Adhering to interventionFig. S11. Risk of bias assessment for the meta-analysis of Observed OTUs - Assignment to intervention; Fig. S12. Risk of bias assessment for the meta-analysis of Observed OTUs - Adhering to intervention. [file 12916_2023_2961_MOESM3_ESM.zip › Fig. S9R2.png]
